# Daily singing of adult songbirds functions to maintain song performance independently of auditory feedback and age

**DOI:** 10.1038/s42003-024-06311-5

**Published:** 2024-05-18

**Authors:** Daisuke Mizuguchi, Miguel Sánchez-Valpuesta, Yunbok Kim, Ednei B. dos Santos, HiJee Kang, Chihiro Mori, Kazuhiro Wada, Satoshi Kojima

**Affiliations:** 1 Sensory and Motor Systems Research Group, Korea Brain Research Institute, Daegu, 41062 Republic of Korea; 2 Department of Life Sciences, Graduate School of Arts and Sciences, University of Tokyo, Tokyo, 153-0041 Japan; 3 Department of Biological Sciences, Faculty of Science, Hokkaido University, Sapporo, 060-0810 Japan; 4 Present Address: Department of Biomedical Engineering, School of Medicine, Johns Hopkins University, Baltimore, MD 21205 USA; 5 Present Address: Faculty of Pharmaceutical Sciences, Department of Life and Health Sciences, Teikyo University, Tokyo, 173-8605 Japan

**Keywords:** Birdsong, Animal behaviour

## Abstract

Many songbirds learn to produce songs through vocal practice in early life and continue to sing daily throughout their lifetime. While it is well-known that adult songbirds sing as part of their mating rituals, the functions of singing behavior outside of reproductive contexts remain unclear. Here, we investigated this issue in adult male zebra finches by suppressing their daily singing for two weeks and examining the effects on song performance. We found that singing suppression decreased the pitch, amplitude, and duration of songs, and that those song features substantially recovered through subsequent free singing. These reversible song changes were not dependent on auditory feedback or the age of the birds, contrasting with the adult song plasticity that has been reported previously. These results demonstrate that adult song structure is not stable without daily singing, and suggest that adult songbirds maintain song performance by preventing song changes through physical act of daily singing throughout their life. Such daily singing likely functions as vocal training to maintain the song production system in optimal conditions for song performance in reproductive contexts, similar to how human singers and athletes practice daily to maintain their performance.

## Abstract (web summary)

This study demonstrates that adult zebra finches maintain song performance by preventing song changes through physical act of daily singing throughout their life.

## Introduction

Singing behavior is a highly complex motor skill that has been extensively studied across many taxa, including birds, mice, whales, and primates including humans^1^. In songbirds, the primary functions of singing are mate attraction and territorial defense^2^. Singing behavior in reproductive contexts is often observed in the presence of apparent recipients, and thus the purpose of singing can be easily determined by the response of the recipients. In contrast, the functions of singing outside reproductive contexts, produced even when no apparent recipients are around, are much less understood. Although this type of singing behavior has been suggested to play important roles in vocal learning and song structure maintenance^3–5^, the exact function of singing outside reproductive contexts remains controversial^6–9^.

**Fig. 1:**
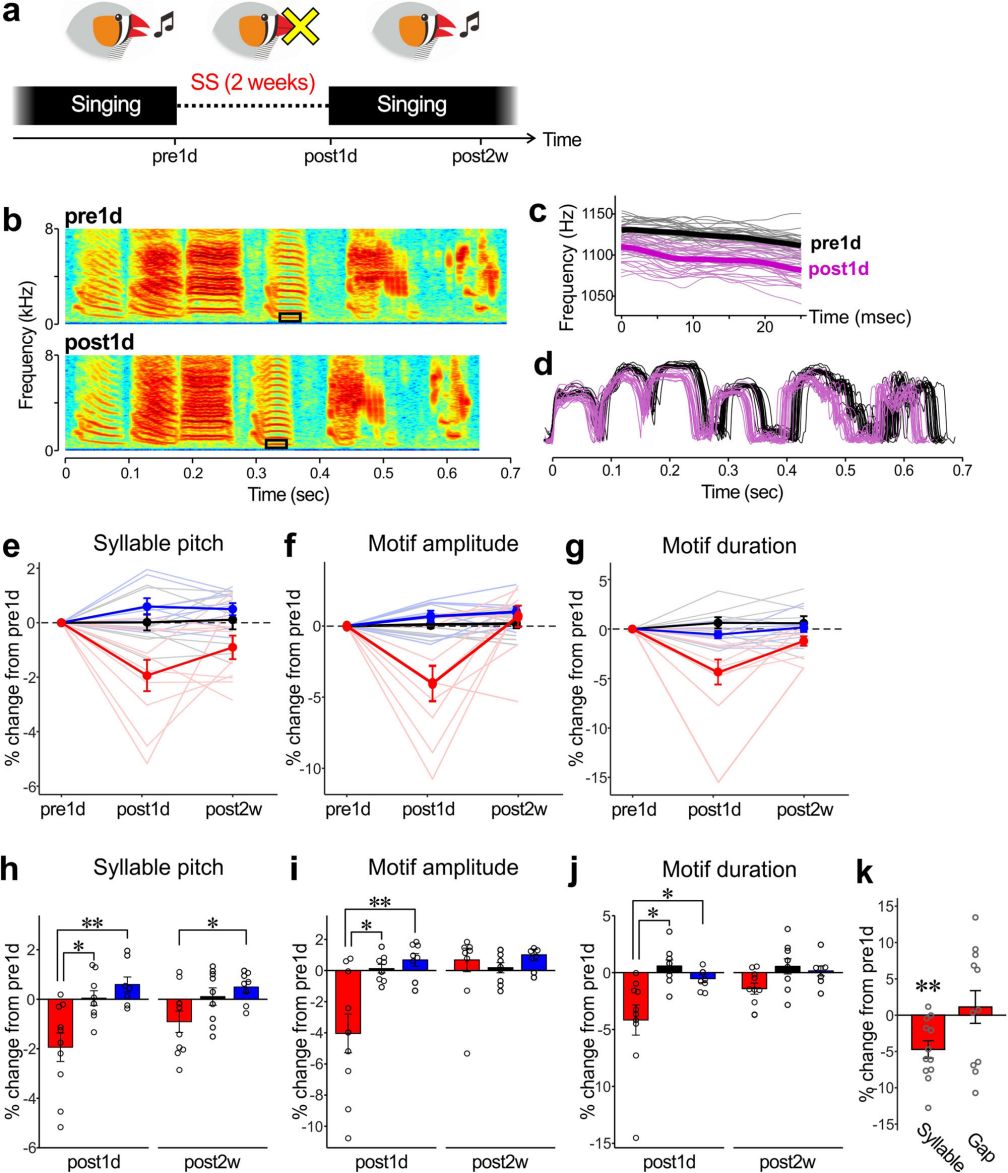
Changes in song performance by suppression of daily undirected singing. **a** Schematic diagram of the experimental procedure. Undirected singing was suppressed for 2 weeks (singing suppression, SS). Songs recorded one day before the onset of SS (pre1d), one day after the end of SS (post1d), and 2 weeks after the end of SS (post2w) were analyzed. **b** Example spectrograms of undirected song motifs at pre1d and at post1d, showing that the overall song structure was maintained. **c** Pitch trajectories of the rectangle regions shown in **b**. Thin lines indicate pitch trajectories from a single syllable rendition; thick lines indicate mean trajectories across all renditions analyzed; black and magenta lines indicate pre1d and post1d songs, respectively. **d** Overlaid amplitude envelopes of song motifs obtained from the bird shown in **b**. Conversions are as in **c**. **e**–**g** Percent changes in syllable pitch (**e**), motif amplitude (**f**), and motif duration (**g**) of SS-treated (red lines), intact control (black lines), and night-weight control (blue lines) birds at post1d and post2w relative to pre1d songs. Thin- and light-colored lines indicate the data of individual birds and thick- and dark-colored lines indicate the mean and SEM across birds. **h**–**j** Post-hoc multiple comparisons of percent changes in song features for post1d and post2w songs. Mean and SEM across birds are shown for SS-treated (red), intact control (black), and night-weight control (blue) birds. All the song features significantly decreased at post1d in SS birds compared to those in control birds and substantially recovered at post2w (**p < 0.01, *p < 0.05). **k** Percent changes (mean and SEM) in syllable and silent gap duration of SS-treated birds at post1d relative to pre1d data. Syllable duration but not gap duration significantly changed after SS procedure (**p < 0.01).

The zebra finch (*Taeniopygia guttata*) is a non-territorial songbird commonly used for neuroethological studies of birdsong. Male zebra finches learn to produce a song during a limited period in early life (closed-ended learner^10^). As adults ( > 90 days post-hatch [dph]) they repeatedly produce thousands of renditions of the learned song daily throughout life, even when isolated from other birds (i.e., outside reproductive contexts)^11–13^. This daily singing in the solo context, generally called “undirected singing”, has been shown to have a function to maintain song structure: through undirected singing, birds evaluate the song structure using auditory feedback (AF) and refine it if necessary^3,4,14–17^ (although other functions of undirected song have been suggested^7–9^). This AF-dependent function of undirected singing, however, appears to be limited to young adult birds, as the removal of AF causes substantial changes in song structure in relatively young birds but not in old birds^16,17^. Nevertheless, old birds still sing many renditions of undirected songs every day^12,18^, raising the possibility that undirected singing serves another function in addition to the AF-dependent maintenance of song structure^19^. To explore novel functions of daily undirected singing, we suppressed undirected singing in adult zebra finches for two weeks (Fig. 1a) using a method to physically interfere with their singing posture^20^ and examined how their song performance was affected. Our results show significant changes in song performance after two weeks of singing suppression (SS) independent of the age of the birds. These song changes were largely recovered through subsequent free singing within a few weeks in both intact hearing birds and deafened birds. These results provide evidence that daily undirected singing functions to maintain song performance throughout life independently of AF.

## Results

### Suppression of daily undirected singing for two weeks alters song performance

We first suppressed daily undirected singing of young adult zebra finches (100-123 dph, n = 10 birds) by attaching weights on their necks to interfere with their singing postures during daytime for two weeks^20^ (Fig. 1a; see Materials and Methods). This manipulation led to a dramatic decrease in the production of undirected songs in all examined birds, reducing it to levels below 10% of the song production observed before the SS period (Supplementary Table 1). To examine the effect of SS on song performance, we compared songs of the SS-treated birds with those of two control groups with similar ages: birds that had weights on their necks only during nighttime and were allowed to sing freely during daytime for two weeks (night-weight control, 103-108 dph, n = 8 birds), and birds that did not have weights at any time for two weeks (intact control, 96-129 dph, n = 9 birds).

We found that, although the SS manipulation did not affect the overall acoustic structure of song motifs (a stereotyped sequence of syllables) (Fig. 1b), it caused significant changes in song performance (Supplementary Audio 1–4 and Supplementary Data): compared with intact and night-weight control birds, SS-treated birds showed significantly greater decreases in the pitch of harmonic syllables (F_2,24_ = 11.21, *p* = <0.001; post1d in Fig. 1c, e, and h; repeated measures two-way ANOVA and post-hoc Dunnett’s multiple comparisons tests; see Table 1 for more details). Moreover, significant decreases were observed in both the root mean square (RMS) amplitude of song motifs (F_2,24_ = 6.312, *p* = 0.006; post1d in Fig. 1d, f, and i), and the mean duration of song motifs (F_2,24_ = 8.715, *p* = 0.001; post1d in Fig. 1d, g, and j). It is important to note that song changes of night-weight control birds were not significantly different from those of intact control birds (Fig. 1e–j and Table 1). Since night-weight birds had weights attached for similar time periods as SS-treated birds (but only during the night), these results indicate that the song changes observed in the SS-treated birds were not due to direct effects of weight attachment, such as long-term posture changes and chronic damages to the vocal production system. Instead, they suggest that these song changes were mainly caused by the reduction of daily undirected singing, and provide evidence that neither the syllable nor motif structures of adult songs are stable without daily undirected singing.

**Table 1:** The results of repeated measures two-way ANOVAs analysis for the data in Fig. 1e–j (SS alone) and those in Fig. 4b–g (SS and deafening)

	Treatment			Time			Interaction		
	F_df_	*p*	*ηp*^2^	F_df_	*p*	*ηp*^2^	F_df_	*p*	*ηp*^2^
**SS alone**									
*Syllable pitch*	F_2,24_ = 11.21	**<0.001**	0.38	F_1.8,42.1_ = 1.575	0.220	0.06	F_4,48_ = 4.757	**0.003**	0.28
*Motif amplitude*	F_2,24_ = 6.312	**0.006**	0.23	F_1.5,36.4_ = 6.747	**0.006**	0.22	F_4,48_ = 7.288	**<0.001**	0.38
*Motif duration*	F_2,24_ = 8.715	**0.001**	0.36	F_1.5,37_ = 4.894	0.020*	0.17	F_4,48_ = 5.018	**0.002**	0.29
**SS & Deaf**									
*Syllable pitch*	F_2,20_ = 2.583	0.101	0.21	F_2,39.3_ = 3.726	**0.034**	0.16	F_4,40_ = 4.796	**0.003**	0.32
*Motif amplitude*	F_2,20_ = 5.045	**0.017**	0.37	F_1.9,37.3_ = 9.481	**<0.001**	0.32	F_4,40_ = 9.002	**<0.001**	0.47
*Motif duration*	F_2,20_ = 0.4273	0.658	0.02	F_1.1,21.8_ = 2.648	0.116	0.12	F_4,40_ = 1.884	0.132	0.16

The bold values indicate *p*-values below the significance level (0.05).

Given a previous study suggesting that syllable pitch and amplitude are regulated by common neural circuitry^21^, we also examined whether the effects of SS are correlated across different song features. Upon examining correlations of SS-induced changes for all possible pairs of the song features examined, we found significant correlations between motif amplitude and motif duration (Supplementary Fig. 1a), but not between syllable pitch and motif amplitude or between syllable pitch and motif duration (Supplementary Fig. 1b, c, respectively). These results may reflect the different timescales between syllable pitch and the other song features (motif amplitude and duration).

### Song performance gradually degrades during the SS period

**Fig. 2:**
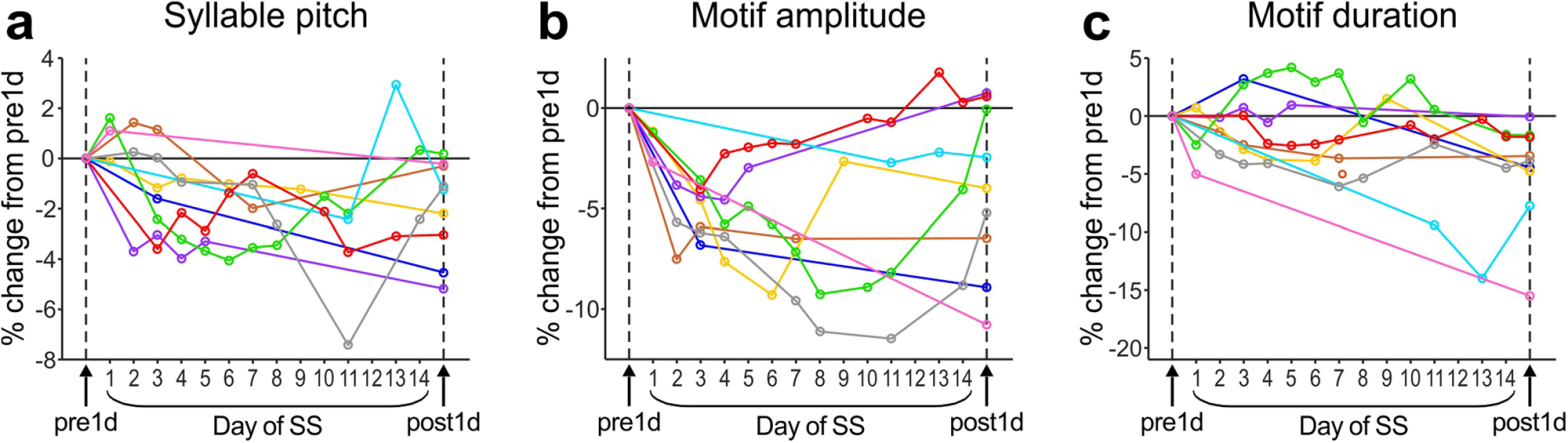
Chronological changes in song performance during the SS period in SS-treated birds. Daily averages of percent changes relative to pre1d songs in syllable pitch (**a**), motif amplitude (**b**), and motif duration (**c**) are shown. The colors indicate individual birds. Note that the number of songs and the timing of song production during the SS period are highly variable across birds (see Supplementary Table 1).

During the SS period, many SS-treated birds produced a small number of songs despite being attached with weights on their necks (Supplementary Table 1). We analyzed these songs to assess the timeframe of song changes during the SS period. We found that both syllable pitch and mean motif duration gradually decreased over multiple days to the levels of post1d songs (Fig. 2a, c and Supplementary Data). These gradual changes provide further evidence supporting that SS-induced changes in those features are predominantly caused by the reduction of daily singing rather than the direct effects of the SS manipulation (weight attachment), such as posture changes. In contrast to syllable pitch and motif duration, motif amplitude decreased more rapidly, even on the first day of song production during the SS period in all birds, and continued to decrease to levels even below the post1d song levels in some birds (Fig. 2b and Supplementary Data). These rapid and substantial decreases in motif amplitude are likely, at least in part, the direct effects of the SS manipulation, such as posture changes. However, considering the results of night-weight control birds (post1d in Fig. 1f & i), which were similar to those of intact control birds, it is unlikely that these direct effects of SS manipulation persisted beyond the SS period or substantially contributed to the amplitude decreases observed in post1d songs. In support of this view, the night-weight control birds showed no significant effects of overnight weight attachment on song performance produced on the following day (comparison between pre1d and the following day, which were intervened by the first night of weight attachment; Supplementary Fig. 2a, p = 0.38, 0.84, and 0.74 for syllable pitch, motif amplitude, and motif duration, respectively, Wilcoxon signed-rank [WSR] test, n = 8 birds). Thus, although the songs that birds managed to produce under the physically restrained condition have abnormally small amplitude, the amplitude decreases observed in post1d songs were not due to direct effects of weight attachment, but instead were mainly attributable to the reduction of daily undirected singing.

Additionally, we examined whether the SS manipulation caused abnormal truncation of song motifs, by comparing the probability of the most typical syllable located at the end of individual song bouts between pre1d songs and songs produced during the SS period (a song bout is defined as one or more repetitions of song motifs, separated from other song bouts by >0.5 sec silent intervals; see Materials and Methods for more detail). There were no significant changes in the probability of the most typical syllable between these songs (Supplementary Fig. 2b, p = 0.13, WSR test, n = 9 birds), providing no indication of abnormal truncation of song motifs during the SS period.

Given the conclusion that song changes observed in the SS-treated birds are mainly attributed to the reduction of daily undirected singing, we wondered if the degrees of song changes were correlated with the number of songs produced during the SS period. However, no significant correlations were observed between song changes at post1d and the number of songs during the SS period for all three song features (Supplementary Fig. 3). These results may reflect the relatively small number of songs produced during the SS period compared to the song amount before SS (i.e., almost complete suppression of daily singing) in all birds and resulting small variations of song amounts across birds.

We also conducted a more detailed analysis of the SS-induced decreases in motif duration by examining the relative contributions of individual syllables and inter-syllable silent gaps to the motif duration decreases. During the late phase of song development, it has been shown that motif duration gradually decreases due almost entirely to a shortening of silent gaps but not of syllables^22^. In sharp contrast, the SS-treated birds showed significant decreases in syllable duration but not in gap duration (Fig. 1k; *p* = 0.0024 and *W* = 75 for syllable duration and *p* = 0.57 and *W* = 31 for gap duration, WSR test). These results indicate that the SS-induced decrease in motif duration was predominantly attributable to effects on the expiratory phase of song production (syllables production) but not on the inspiratory phase (silent gaps)^23^.

### Song performance substantially recovers through daily singing even without auditory feedback

**Fig. 3:**
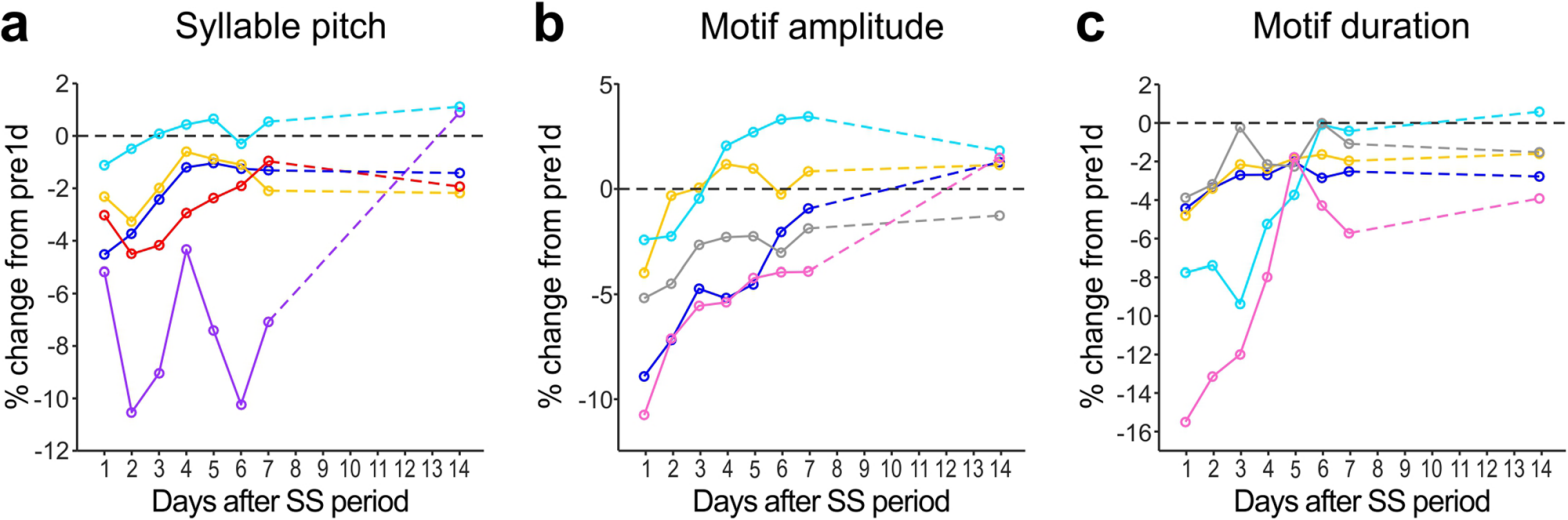
Time courses of recovery of song performance after SS period. For syllable pitch (**a**), motif amplitude (**b**), and motif duration (**c**), daily averages of percent changes relative to pre1d songs are shown from post1d to post7d as well as at post14d in the birds that exhibited substantial song changes at post1d. The colors correspond to the birds shown in Fig. 2a–c.

In SS-treated birds, the altered syllable and motif structures tended to return to their pre-SS levels and became similar to the songs of intact and night weight control birds after 2 weeks of free singing following the SS period (post2w in Fig. 1e–j; for syllable pitch, *p* = 0.152 vs. intact and *p* = 0.023 vs. night weight controls, post-hoc Dunnett’s multiple comparisons test; for motif amplitude, *p* = 0.777 vs. intact and *p* = 0.901 vs. night weight controls; for motif duration, *p* = 0.059 vs. intact and *p* = 0.057 vs. night weight controls). To evaluate the time courses of song recovery in better detail, we analyzed the features of songs produced over the seven days following the SS period (i.e., from post1d to post7d) in the birds that exhibited substantial song changes at post1d (Fig. 3a–c and Supplementary Data). We found that all song features recovered over multiple days and mostly plateaued around post7d, reaching values similar to post14d, although there were large variations in recovery speed across song features and across birds (Fig. 3a–c). These reversible effects of SS on song performance suggest that daily undirected singing has a function to prevent song changes that can occur in the absence of singing and to maintain song performance in an optimal condition.

**Fig. 4:**
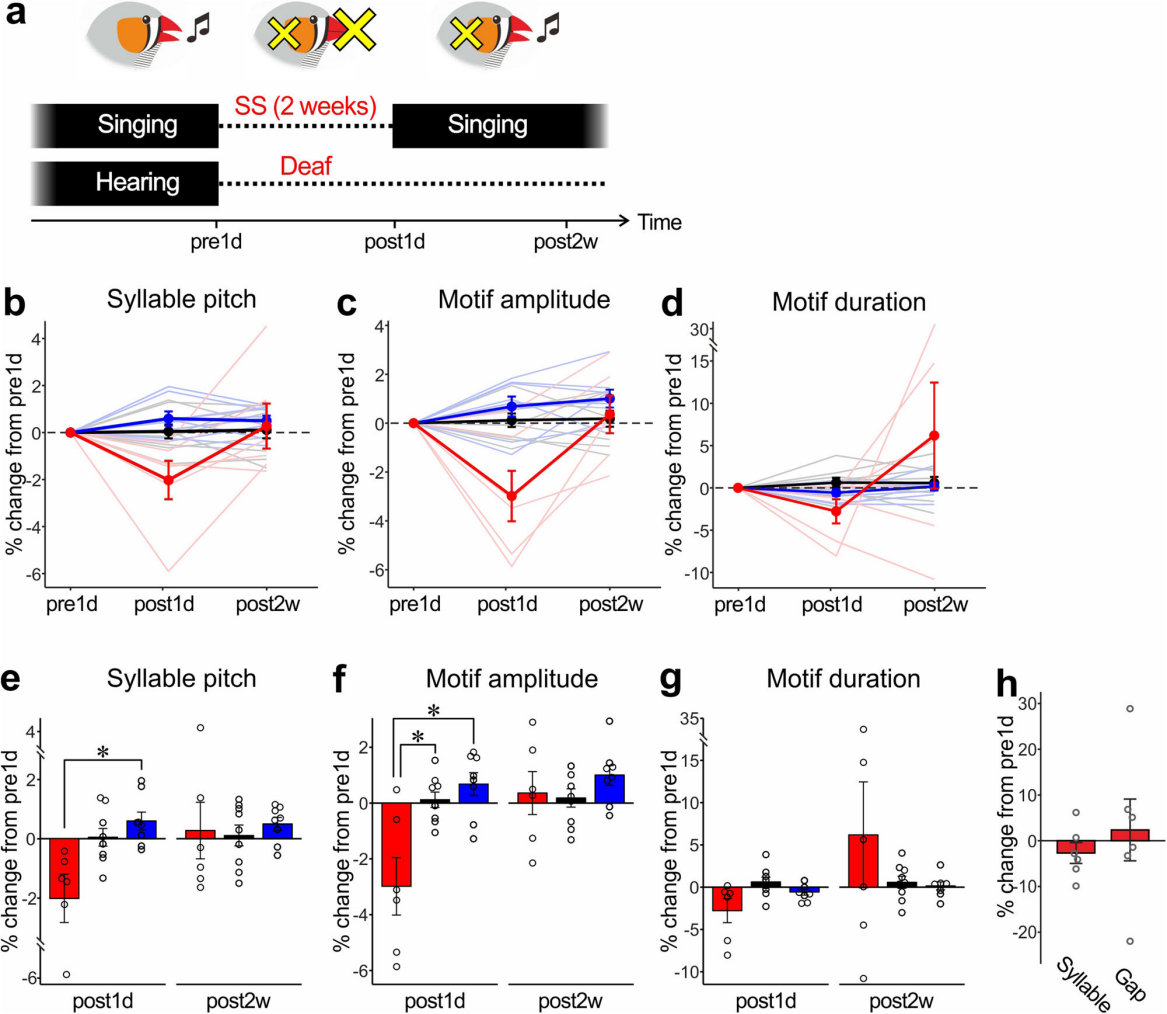
AF-independent recovery in song performance after SS through daily undirected singing. **a** Schematic diagram of the experimental procedure. Birds were deafened immediately before SS. **b**–**d** Percent changes in syllable pitch (**b**), motif amplitude (**c**), and motif duration (**c**) in deafened and SS-treated birds (red lines), night-weight control birds (blue lines), and intact control birds (black lines). Conversions are as in Fig. 1e–g. **e**–**g** Post-hoc multiple comparisons of percent changes in song features for post1d and post2w songs. Conversions are as in Fig. 1h–j. *p < 0.05. **h** Percent changes in syllable and silent gap duration of SS-treated birds at post1d relative to pre1d data.

Undirected singing has been previously shown to function in the maintenance of song structure by providing AF, through which they evaluate their own song structure and correct vocal errors^3,4,14,15^. We wondered whether similar, AF-dependent mechanisms mediate the recoveries of song performance after SS. To examine this, we deafened young adult birds (98-101 dph) to remove AF immediately before the SS period and examined whether their song recovery following SS was affected (Fig. 4a and Supplementary Data). Although the overall song structure was maintained after SS in most deafened birds (6 out of 7 birds), they showed reversible changes in syllable pitch and motif amplitude similar to those observed in intact-hearing birds (Fig. 4b–g; for syllable pitch, *p* = 0.088 vs. intact and p = 0.038 vs. night weight controls at post1d, and *p* = 0.979 vs. intact and p = 0.962 vs. night weight controls at post2w; for motif amplitude, *p* = 0.048 vs. intact and p = 0.025 vs. night weight controls at post1d, and *p* = 0.966 vs. intact and p = 0.670 vs. night weight controls at post2w; see Table 1 for the results of repeated measures two-way ANOVA). A similar trend was observed for motif duration as well, although the results were not significantly different (Fig. 4d and g; *p* = 0.108 vs. intact and p = 0.295 vs. night weight controls at post1d, and *p* = 0.597 vs. intact and p = 0.556 vs. night weight controls at post2w). Also, there were trends for decreases in syllable duration and silent gap duration similar to that in intact hearing birds (Fig. 4h), although neither decrease was statistically significant (*p* = 0.44 and *W* = 15 for syllable duration; *p* = 0.69 and *W* = 8 for gap duration, WSR test). Thus, the recovery of song performance after SS does not necessarily require AF, and is likely to be caused mainly by singing behavior per se (i.e., the physical act of singing). These results suggest an AF-independent function of undirected singing to maintain song performance.

### SS-induced song changes do not depend on age

**Fig. 5:**
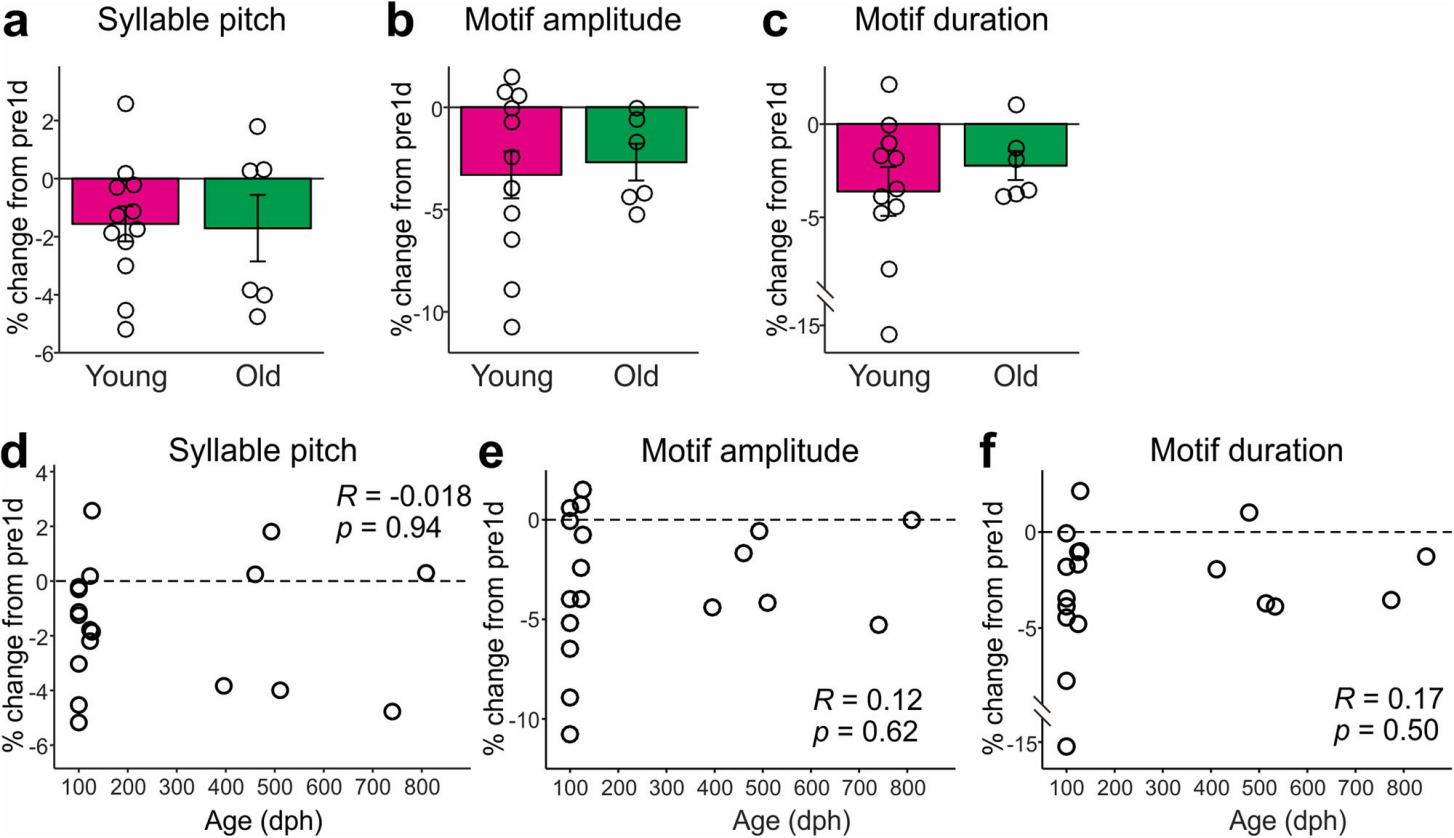
SS-induced changes in song performance do not depend on age. **a**–**c** Comparisons of percent changes at post1d in syllable pitch (**a**), motif amplitude (**b**), and motif duration (**c**) between young adult birds and relatively old adult birds (mean and SEM). There were no significant differences between them (*p* > 0.05). **d**–**f** Percent changes in the song features plotted against the ages of birds. There were no significant correlations between age and the changes in the song features.

In zebra finches, the AF-dependent function of undirected singing to maintain song structure strongly depends on age: the removal of AF causes substantial changes in song structure in young adult birds ( < ~200 dph) but not in older birds^16,17^. Given that old birds continue to sing many renditions of undirected songs every day (although the number of undirect songs decreases with age)^12,18^, it is possible that SS-induced changes in song structure can occur independently of age and that these changes are prevented by the daily undirected singing throughout the bird’s life. To test this possibility, we examined the effects of SS on song structure in relatively old birds with intact hearing (396–809 dph, n = 6 birds) and compared them with the data of young adult birds shown in Fig.1 (100-123 dph, n = 10 birds). We found that SS-induced changes at post1d were not significantly different between young adult birds and old adult birds in any of the song features examined (Fig. 5a–c and Supplementary Data; *p* = 0.89 for syllable pitch, *p* = 0.96 for motif amplitude, *p* = 0.82 for motif duration, Mann-Whitney U test). Also, when we combined the data of young adult birds and those of old adult birds, there were no significant correlations between SS-induced song changes and age (Fig. 5d–f; *R* = −0.018, *p* = 0.94 for syllable pitch; *R* = 0.12, *p* = 0.62 for motif amplitude; *R* = 0.17, *p* = 0.50 for motif duration). These results demonstrate that, unlike the AF-dependent song plasticity previously reported^16,17^, SS-induced song changes do not depend on age, suggesting that birds produce daily undirected songs to maintain song structure throughout their life.

### SS-induced song changes are not explained by stress or bodyweight loss

**Fig. 6:**
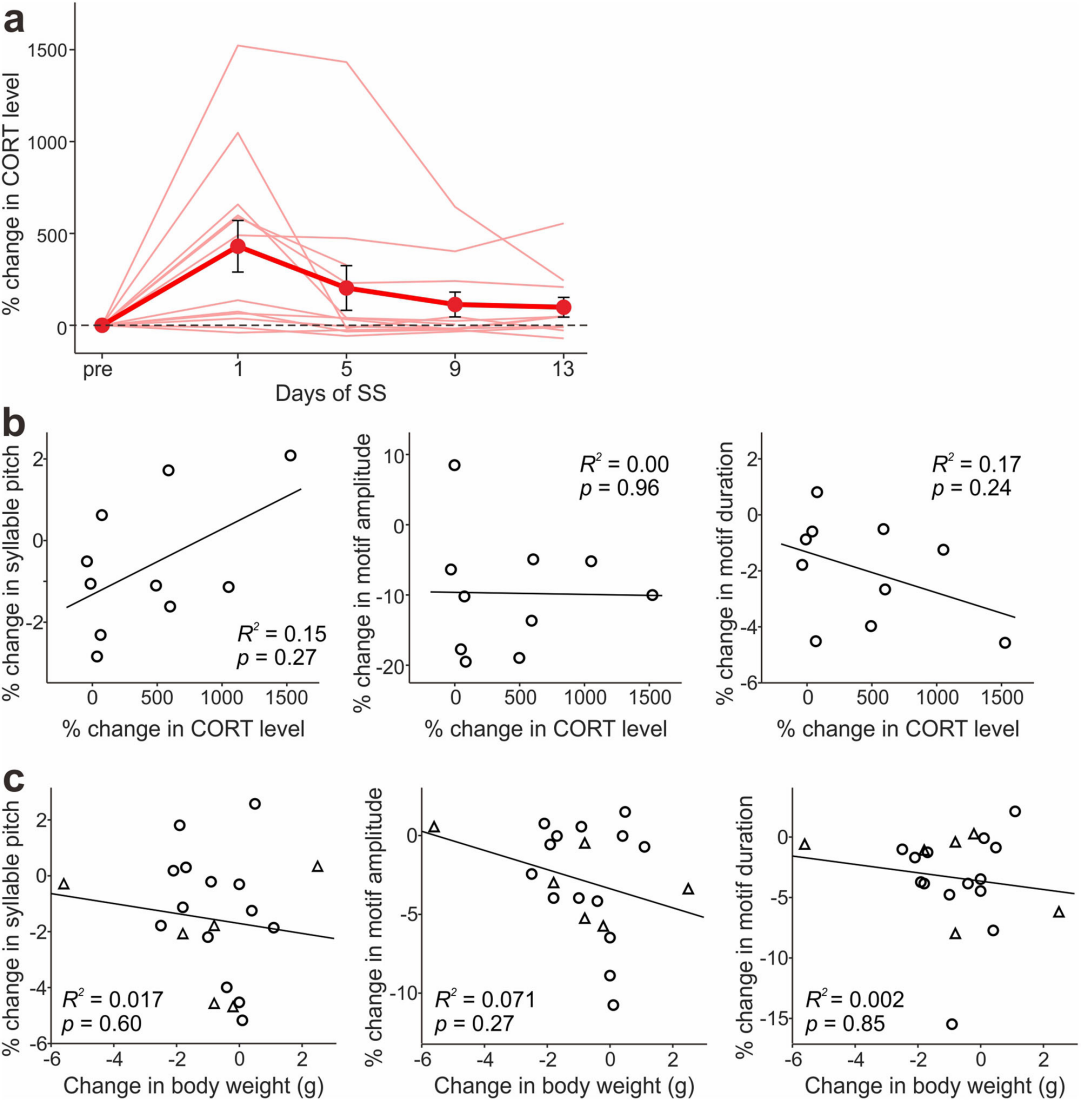
SS-induced changes in song performance are not explained by stress or bodyweight loss. **a** Percent changes in plasma CORT levels caused by SS treatment. Thin lines indicate the data of individual birds and thick lines indicate the mean ( ± SEM) across the birds. **b** Percent changes in syllable pitch (*left*), motif amplitude (*middle*), and motif duration (*right*), plotted against those in CORT levels measured on the 1^st^ day of SS. **c** Percent changes in syllable pitch (*left*), motif amplitude (*middle*), and motif duration (*right*), plotted against bodyweight changes (the song data in Figs. 1 and 4 were replotted). Circles and triangles indicate SS birds with normal hearing and those with hearing impaired (deafened), respectively.

Because our SS procedure is likely to induce substantial physical stress in the birds, we cannot exclude the possibility that the song changes observed after the SS period were predominantly caused by the physical stress induced by the SS procedure rather than the reduction of undirected singing. To examine this possibility, we measured plasma corticosterone (CORT) levels in a different group of birds before and during the SS period (see Methods for more detail). We found that many birds increased CORT levels on the first day of the SS period to levels similar to those induced by restraint stress reported in previous studies^24,25^ and subsequently decreased toward the pre-SS levels (Fig. 6a and Supplementary Data; *n* = 10 birds). Also, the increases in CORT levels on the first day of the SS period were not significantly correlated with SS-induced changes in the song features that we examined (Fig. 6b; *n* = 10 birds; see Supplementary Table 4 for detailed statistical results). These results support the notion that SS-induced song changes are mainly attributed to the reduction of undirected singing rather than the physical stress caused by SS procedure. Moreover, although many birds that received SS exhibited weight loss, an indirect indicator of stress level, no significant correlations were found between the weight changes and the song changes (Fig. 6c and Supplementary Data; the song data in Figs. 1 & 2 were replotted to be compared with weight changes; see Supplementary Table 4 for detailed statistical results), further supporting the independence of SS-induced song changes from physical stress. In summary, the song changes induced by SS are most likely caused by the reduction of singing behavior, rather than stress.

### SS-treated birds decreased daily amounts of undirected singing immediately after the SS period

A previous study showed that short-term suppression of undirected singing (for 5-10 h during the daytime) in adult zebra finches induces intense singing immediately after the SS period, indicating that intrinsic motivation for undirected singing increases while singing is suppressed^26^. We examined whether a similar increase in singing motivation is caused by the SS manipulation that we used by comparing daily amounts of undirected song bouts between pre1d and post1d in SS-treated birds (100-123 dph, the same birds used for Figs. 1–3). We found a significant decrease in daily singing amounts in post1d compared to pre1d (Supplementary Fig. 4, p = 0.037, WSR test, n = 10 birds), suggesting decreasing effects of our long-term SS manipulation on intrinsic singing motivation. These results contrast with the increasing effects of short-term SS on singing motivation previously reported^26^.

## Discussion

In the present study, we demonstrate that the suppression of daily undirected singing in adult zebra finches induces gradual changes in both syllable and motif structures of their songs. Notably, these changes can be recovered within a short period of free singing. These results suggest that undirected singing may function in preventing such song changes and in maintaining song performance at an optimal level. Moreover, we show that the recovery of song performance following the SS period does not necessarily require AF, highlighting the importance of the physical act of singing in the maintenance of adult song performance. Finally, in contrast to adult song plasticity reported in previous studies^16,17^, our results indicate that song changes induced by SS can occur even in relatively old birds, suggesting no strong dependence of the song maintenance mechanisms on age. Taken together, our findings provide evidence suggesting that zebra finches maintain their song performance by preventing age-independent song changes through the physical act of daily singing throughout life. Because male zebra finches produce courtship songs with a motif structure very similar to that of undirected song^10^ (although variability and speed are slightly different), it is likely that they maintain the song production system in an optimal condition through undirected singing to prepare for the best song performance in reproductive contexts^19^.

The critical role of undirected singing as motor practice for song maintenance has been already suggested by a number of previous studies, and the detailed mechanisms have been intensively studied. Accumulating evidence suggests that during undirected singing but not female-directed singing, birds actively generate variability in song structure to explore vocal space and evaluate ongoing song performance through AF to acquire motor commands that leads to improved performance^4,27,28^. These song maintenance mechanisms have also been well studied at the neural circuit level and extensive knowledge has been accumulated^29–31^. The song maintenance mechanisms that we revealed in the current study are clearly different from those AF-dependent mechanisms. First, it does not strongly depend on AF and thus is unlikely to require evaluation of song acoustic structure, although they may require proprioceptive feedback to evaluate the motor commands and/or muscle activities. Also, the AF-dependent mechanisms depend strongly on age, with significant age-dependent declines of both exploratory song variability and AF-dependent song plasticity^16,17,32^, whereas the novel mechanisms do not show strong age dependence. Thus, adult birds appear to maintain the song structure using both AF-dependent and independent mechanisms when they are relatively young, and subsequently decrease the contribution of the AF-dependent mechanisms while largely maintaining the AF-independent mechanisms throughout their life. These findings provide a plausible explanation for why many renditions of undirected song are produced even in old age, although other functions of undirected song have also been suggested^7–9^.

A previous study showed that temporary suppression of undirected singing (for 5-10 h during the daytime) in adult zebra finches induces intense singing immediately after the SS period, indicating that intrinsic motivation for undirected singing increases while singing is suppressed^26^. This SS-induced increase in singing motivation and resulting intense singing can be explained by the song maintenance function of undirected singing that we found in the present study. It is reasonable to assume that birds produce intense singing after temporary SS to compensate for the loss of vocal practice during the SS and to quickly re-optimize the vocal system and song structure for future courtship activity, just as professional singers and speakers warming up their voices before performing to optimize their vocal quality^33^. Consistent with this idea, substantial song deterioration after non-singing periods and improvement during subsequent singing are observed in juvenile birds producing immature songs^11,13^. Even in adult birds, subtle changes in song microstructure are observed primarily during the night^34^, in which zebra finches do not usually sing. It remains to be elucidated, however, temporary SS during daytime causes any changes in adult song structure and recovers through subsequent daily singing. It is also worth noting that, in contrast with intense singing immediately after short-term SS previously reported^26^, we found a significant decrease in daily song amount after two weeks of SS (Fig. 2h). This discrepancy is likely to be attributable to the great difference in the time course of the experiments between the two studies. In Kim et al. ^26^, SS periods were relatively short (up to 10 h) and ended in daytime hours, and undirected singing behavior was examined immediately after the SS periods on the same day. In contrast, we suppressed undirected singing all day (entire daytime period) for 2 weeks and then examined undirected singing behavior on the following day. Because unlike daytime lights-out that causes SS^26^, the nighttime dark period and resulting nighttime SS does not dramatically increase singing motivation in the next morning, it is very likely that enhanced singing motivation caused by daytime SS is reset by night-time sleep and not maintained overnight into the next day. Additionally, it is possible that our long-term SS manipulation gradually decreases undirected singing motivation over days due to some physiological factors including weakening of the respiratory system responsible for song production caused by the SS manipulation.

The SS manipulation dramatically decreased the number of songs recorded during the SS period (Supplementary Tables 1–3). Although our song recording system was set up to capture sounds that were much quieter than normal zebra finch songs, we cannot exclude the possibility that the birds produced songs whose volumes were even below the threshold of our recording system during the SS period. Similarly, we cannot exclude the possibility that the birds produced songs with abnormally short durations or other adjustments that were not detected by our recording system (although we did not find any indications of abnormal motif truncations in the songs recorded during the SS period [Supplementary Fig. 2b]). Even if these are the case, however, our results still indicate that the SS manipulation mostly suppressed the production of songs that our recording system can detect and resulted in substantial changes in the structure of such detectable songs after the SS period. These findings provide evidence that production of such detectable songs is required for maintaining their structure, and thus still of great significance in understanding the function of daily undirected singing.

Our results indicating that the long-term SS induces substantial changes in adult song structure and amplitude are largely consistent with the findings of a recent study by Adam et al. ^19^. Although they employed a different SS method, both our results and theirs lead to virtually the same conclusion. However, our study includes the following new findings that were not reported in Adam et al. ^19^: SS-induced song changes are mostly recovered within a short period of free singing, providing more direct evidence for the function of undirected singing in maintaining adult song performance; the recovery of song performance following the SS period does not necessarily require AF, revealing novel, AF-independent mechanisms for the maintenance of song performance; SS-induced song changes do not strongly depend on age, highly contrasting with AF-dependent song plasticity that has previously reported^16,17^. Because Adam et al. ^19^ also reported the experimental findings that are not included in our current study, these two studies complement each other to significantly advance our understanding of the mechanisms by which adult birds maintain song performance through daily undirected singing.

Adam et al. ^19^ examined the physiological mechanisms underlying SS-induced changes in song performance. They reported evidence that SS-induced song changes are caused, at least in part, by physiological and anatomical changes in the syringeal muscles responsible for song production^19^, similar to the atrophy of skeletal muscles that is generally known to be caused by a lack of physical activity. It is also possible, however, that SS-induced song changes are attributable to changes in the neural circuits that generate song structure. Passive drift of micro-circuits in the vocal motor pathway is likely to occur during the SS period, but changes in those circuits could be actively caused by the input from the anterior forebrain pathway, which actively promotes song plasticity in freely singing birds^15,35–37^. In accord with the idea of neural origin of SS-induced song changes, substantial changes in singing-related neural activity in the song motor pathway occur primarily during the nighttime (when birds do not sing), and less frequently during the daytime^34,38^. Also, changes in motif duration are observed even in birds that freely sing when the central neural circuits are manipulated^39,40^, further supporting the idea of neural origin of SS-induced song changes. To directly examine the contribution of neural circuit changes to SS-induced song changes, it would be necessary to record song premotor activity before and after the SS period from the same neurons and compare the activity patterns. For such experiments, chronic calcium imaging of song premotor activity in a population of neurons would be required. Regardless of the mechanisms of song changes induced by the lack of daily undirected singing, our findings open the possibility that the physical activity-dependent and hearing-independent mechanisms for maintaining vocal performance are commonly used in many other bird species and other animal taxa, including non-vocal learning birds and mammals.

## Materials and methods

### Subjects

Adult male zebra finches (*Taeniopygia guttata*, 96–809 dph) were bred in our colony at our institute and individually housed in sound-attenuating chambers (MC-050, Muromachi Kikai, or custom-made ones) on a 14:10-h light:dark cycle throughout the experiments. Their care and treatment were reviewed and approved by the Institutional Animal Care and Use Committee (IACUC) at the Korea Brain Research Institute. All experiments were performed in accordance with relevant guidelines and regulations.

### Song recording

Songs were recorded using a microphone (PRO35, Audio-Technica) positioned 5 ~ 10 cm above the cage (20 ×20 x 20 cm) and a custom-made recording program written in Java at a 32-kHz sampling rate with 16-bit resolution. To minimize the influence of the bird’s position on the amplitude of song recording, no perch was placed in the cage and the food was provided in a petri dish placed on the bottom of the cage so as to have the bird sing at the cage bottom (zebra finches rarely sing while holding on to the side walls or ceiling of the cage). Song recording was triggered when the program detected 4 or 5 successive song elements, each of which reached predefined thresholds of amplitude and duration, and ended when a silent period lasted >0.5 sec (amplitude thresholds were set to detect as many vocalizations as possible and to ensure that background noise was not frequently recorded). Our recording system thus captured mostly the song bouts (one or more repetitions of a song motif, which consists of a stereotyped sequence of several syllables), although non-song sounds such as short calls and cage noises were occasionally recorded. Birds that produced songs containing at least one harmonic syllable with sufficient singing rates ( > 200 undirected song bouts per day) were used for experimentation.

### Singing suppression

Spontaneous undirected singing was suppressed for two weeks using a previously published method^20^. Briefly, a custom-made weight (20.1-32.6 g) was attached to the neck of the bird each morning to interfere with his singing posture to prevent him from singing (the weight shifted the posture toward an inferior position). The weight was adjusted daily for each bird so that the procedure mostly suppressed undirected singing but did not severely affect the daily behaviors of the bird, such as eating, drinking, preening, and calling (body weight and health conditions were carefully monitored every day). To avoid possible chronic effects of posture manipulation by the weight on the song production system (pain, etc.), the weight was removed every evening when the light was turned off; no singing behavior was observed during the night. With this method, the daily singing amount (the number of song bouts per day) decreased to less than 10% of that of pre-SS (see Supplementary Tables 1–3). In addition to the SS-treated birds, we also had the following two control groups: birds that had weights on their necks only during nighttime and were allowed to sing freely during daytime for two weeks (night-weight control), and birds that did not had weights at any time (intact control).

### Song analysis

To examine the effects of SS on song performance, we compared the songs recorded 1 day before SS (pre1d) with songs recorded 1 day and 2 weeks after SS for all birds (post1d and post2w, respectively; Fig. 1a). In a subset of birds, we analyzed the songs recorded daily from post1d to post7d and compared those data with the post1d data. Also, many birds produced small number of songs during the SS period (Supplementary Table 1), and we analyzed them to examine time courses of changes in song performance.

To test the effect of SS on the pitch of individual syllables, >30 motifs from the songs recorded at each time point were randomly selected, and syllables containing clear and relatively flat harmonic structure were extracted (because it is difficult to detect subtle effects on pitch in harmonic segments with large frequency modulation). For each syllable rendition, the pitch trajectory was obtained from the harmonic segment as described in a previous study^27,40^. Briefly, spectrograms were calculated using a Gaussian-windowed short-time Fourier transform (*σ* = 1 ms) sampled at 8 kHz, and a pitch trajectory was obtained by calculating the fundamental frequency in individual time bins. The fundamental frequency was calculated by parabolic interpolation. For each syllable, all renditions of pitch trajectories were aligned by the syllable onsets, which were determined based on amplitude threshold crossings, and the mean pitch values calculated over the flat portions of pitch trajectories were averaged across all renditions. All the birds examined in the present study had at least one syllable with a clear harmonic structure in their song motif; for the birds that had more than one harmonic syllable, we calculated the effects of SS (percent changes from pre1d) on the pitch of individual syllables and averaged them across the syllables for each bird (the mean number of syllables examined per bird = 1.76; max = 3).

For the amplitude measurements, 12-50 motifs with low background noise levels were randomly selected from the songs recorded at each time point for each bird. We then measured the RMS amplitude values for individual motifs and averaged them. Since we were only interested in SS-induced changes in the motif amplitude, we did not calculate the absolute amplitude and instead obtained percent changes relative to the amplitude of pre1d song. Because our cage setting limited birds’ singing sites to the bottom of the cage (see the description of song recording above), the distance between a singing bird and the microphone did not dramatically change, and therefore changes in the RMS amplitude across different time points are attributed mainly to changes in song volume rather than changes in singing sites.

Durations of song motifs, syllables, and silent gaps were measured by thresholding amplitude envelopes that were normalized to their peak amplitudes (the same thresholds were used for all the time points for each bird). The obtained song segmentations were visually inspected and manually modified if necessary, so that syllable/gap segmentation patterns were identical across all motif renditions. Percent changes in syllable duration or in silent gap duration were calculated for individual syllables or gaps, and then averaged across different syllables or gaps for each bird.

To examine whether the SS manipulation caused abnormal truncation of song motifs, we randomly selected >20 bouts from pre1d songs and songs produced during the SS period, labeled all syllables comprising song motifs, and compared the probability of most typical syllable located at the end of the song bouts between pre1d songs and SS songs. If the probability significantly decreases, it would indicate that birds make abnormal truncation of song motifs during the SS period.

### Deafening

For experiments to test whether AF was related to the song recovery after the SS period, we deafened young adult birds (98-101 dph) immediately before SS by bilateral cochlea removal following a previously published method^5,18,35^ and examined whether song recovery following SS was affected. SS-induced changes in syllable pitch, motif amplitude and motif duration were analyzed in the same way as normal-hearing birds. One bird was excluded from the analysis because the song structure was severely deteriorated by deafening.

### Corticosterone analysis

To measure the stress levels of SS-treated birds, blood samples were collected ( < 75 μl for each sampling) at 5-time points before and during the SS period (pre4d, post1d, post5d, post9d, and post13d) by puncturing the brachial wing vein with a sterile needle (23 G) 1 h after the weight attachment. The blood samples were then centrifuged at 3500 rpm for 20 min at room temperature (approximately 20 °C). The separated serums were stored at −20 °C until the assay. Corticosterone (CORT) levels were measured using an Enzyme Immunoassay kits (Enzo Life Science). Two separate 96-well plates were used in this study, and a separate standard curve was run on each plate. All serum samples (1:20 dilution) and standards were run in duplicates. Plates were read on Absorbance 96 plate reader (Enzo Life Sciences). CORT levels were determined from 4-PL regression of 5 standards ranging from 32 to 20,000 pg/ml. We calculated intra-assay variability as the average %CV between duplicates for each assay and inter-assay variability as %CV among CORT concentrations calculated from each standard curve for 5 corticosterone standards run in each assay. Average intra- and inter-assay variability was 18.0% and 4.8%, respectively.

### Statistics and reproducibility

Statistical tests were performed using Prism 8.4 (GraphPad Software LLC, San Diego CA) and MATLAB R2015b (MathWorks). The effects of SS on syllable pitch and motif amplitude and duration were examined using repeated measures two-way ANOVAs and post-hoc Dunnett’s multiple comparisons tests. Geisser-Greenhouse’s corrections were applied to address sphericity violations. The effects of SS on syllable duration and silent gap duration were examined using Wilcoxon signed-rank tests. The difference in song changes between young adult birds and old birds were examined using Mann-Whitney U tests. The relationships of song changes with age and between different song features were examined Pearson’s correlation coefficients. The relationships of song changes with CORT changes, weight changes, and the number of songs produced during the SS period were examined using multivariate linear regression analyses.

### Supplementary information

Peer review file

Supplementary Information

Description of additional supplementary files

Supplementary Data

Supplementary Audio 1

Supplementary Audio 2

Supplementary Audio 3

Supplementary Audio 4

## Data Availability

The authors declare that the data supporting the findings of this study are available within the paper and its Supplementary Information. Should any raw data files be needed in another format they are available from the corresponding author upon reasonable request.
