# Supplementary material for: Daily singing of adult songbirds functions to maintain song performance independently of auditory feedback and age
Source: Commun Biol. 2024 May 18;7:598. doi: 10.1038/s42003-024-06311-5 (PMC11102546; doi:10.1038/s42003-024-06311-5)
Supplement: Supplementary file 2 — Supplementary Information [file 42003_2024_6311_MOESM2_ESM.pdf]

Supplementary Information

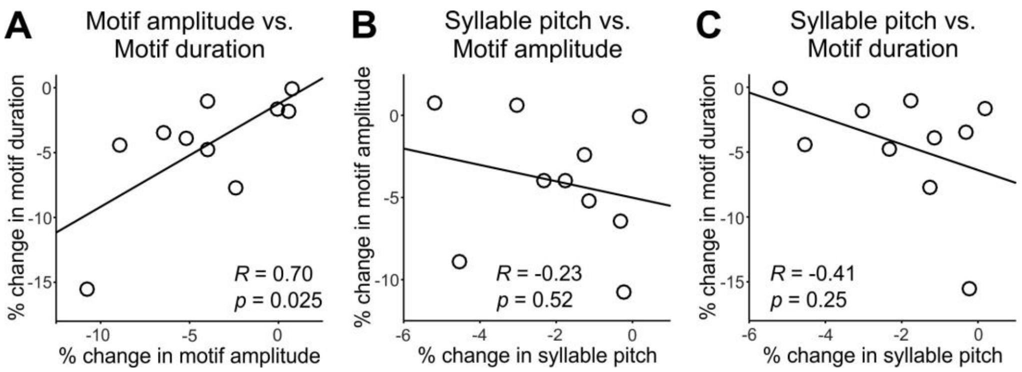

Supplementary Figure 1. Relationships of percent changes at post1d across different song features in SS-treated birds. (A) Motif amplitude vs. motif duration. (B) Syllable pitch vs. motif amplitude. (C) Syllable pitch vs. motif duration.

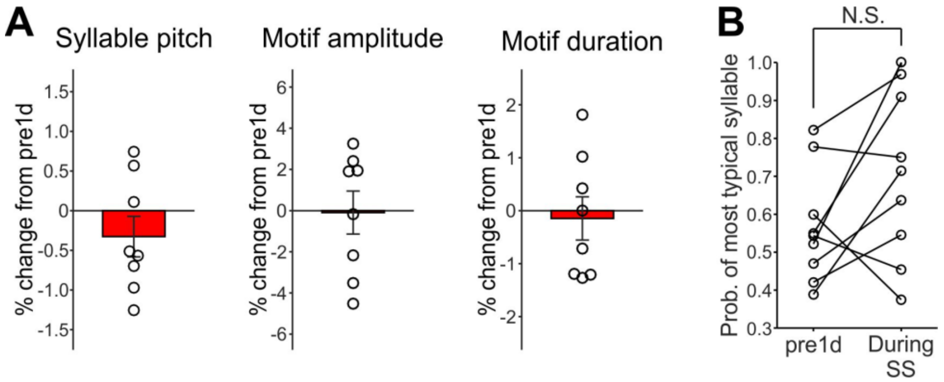

Supplementary Figure 2. (A) Percent changes in the song features in night-weight control birds from pre1d to the following day, intervened by the first night of weight attachment. There were no significant changes in any of the song features ($p > 0.05$ for syllable pitch, motif amplitude, and motif duration). (B) Comparisons of the probability of most typical syllable located at the end of individual song bouts between pre1d songs and songs produced during the SS period in SS-treated birds. There were no significant changes between these songs ($p > 0.05$).

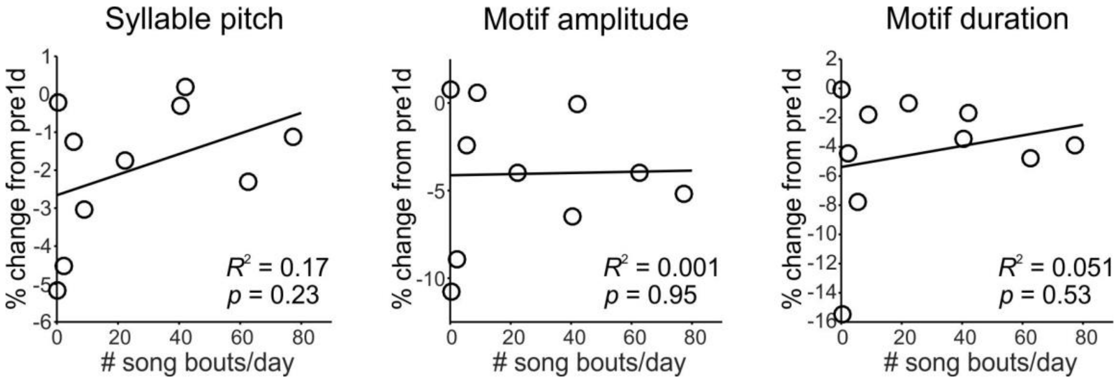

Supplementary Figure 3. Percent changes in the song features at post1d relative to pre1d plotted against the number of songs produced during the SS period.

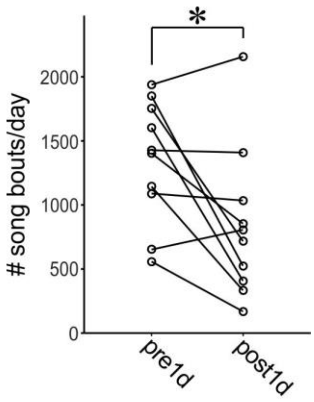

Supplementary Figure 4. Comparison of the daily singing amount (the number of song bouts per day) between pre1d and post1d in SS-treated birds. *$p < 0.05$.

Supplementary Table 1. Daily counts of the number of undirected song bouts before and during SS in young adult birds with intact hearing (the birds used for Figs. 1-3).

**Manipulation**	**SS (intact hearing)**									
**Bird ID**	**b17p6**	**b19r17**	**m67y50**	**y69r26**	**y92w65**	**w78w6**	**m52m41**	**b80p39**	**m56p64**	**w32r84**
**Age (dph)**	100	100	100	100	100	100	100	123	123	123
Before SS										
**pre1d**	1602	1403	558	821	1753	1938	1850	1089	1427	1143
During SS										
**day1**	0	0	69	0	0	0	0	0	0	0
**day2**	31	165	2	447	0	0	0	1	0	4
**day3**	0	284	0	556	0	0	0	121	22	300
**day4**	0	0	0	44	4	0	0	8	4	0
**day5**	0	0	0	0	0	0	0	0	2	3
**day6**	0	116	0	0	1	0	0	246	24	1
**day7**	0	0	0	16	1	0	0	0	2	1
**day8**	0	0	0	17	0	55	0	0	53	0
**day9**	0	0	0	0	0	15	0	500	0	5
**day10**	0	0	0	0	0	15	0	0	3	0
**day11**	0	0	6	1	0	31	0	0	241	0
**day12**	0	0	0	0	0	4	0	0	0	0
**day13**	0	0	1	0	0	2	0	0	0	0
**day14**	0	0	0	0	0	2	0	0	238	0
**Mean during SS (/day)**	2.2	40.4	5.6	77.2	0.4	8.9	0.0	62.6	42.1	22.4
**Relative to pre1d (%)**	0.1	2.9	1.0	9.4	0.0	0.5	0.0	5.7	2.9	2.0

Supplementary Table 2. Daily counts of the number of undirected song bouts before and during SS in deafened birds (the birds used for Fig. 4).

**Manipulation**	**SS & deafened**					
**Bird ID**	**w27k27**	**o87r58**	**k6y57**	**k83w25**	**p1r56**	**y34m86**
**Age (dph)**	98	99	100	100	100	101
Before SS						
**pre1d**	265	1167	1089	1489	2825	1965
During SS						
**day1**	0	0	0	0	0	0
**day2**	0	0	0	0	0	0
**day3**	0	0	0	0	0	0
**day4**	0	0	0	0	16	0
**day5**	31	0	0	0	0	43
**day6**	3	0	0	26	15	0
**day7**	101	3	0	0	0	86
**day8**	33	141	1	1	0	27
**day9**	0	0	1	0	0	0
**day10**	0	0	2	0	0	0
**day11**	0	0	1	0	0	0
**day12**	0	0	3	0	0	0
**day13**	9	0	0	0	0	0
**day14**	2	0	0	0	0	0
**Mean during SS (/day)**	12.8	10.3	0.6	1.9	2.2	11.1
**Relative to pre1d (%)**	4.8	0.9	0.1	0.1	0.1	0.6

Supplementary Table 3. Daily counts of the number of undirected song bouts before and during SS in relatively old birds (the birds used for Fig. 5).

**Manipulation**	**SS (intact hearing)**					
**Bird ID**	**o26b44**	**w51o37**	**r47w46**	**b81k6**	**m5k36**	**y1r60**
**Age (dph)**	396	460	493	511	740	809
Before SS						
**pre1d**	205	734	633	362	536	304
During SS						
**day1**	0	0	0	0	41	0
**day2**	0	1	3	0	4	0
**day3**	19	0	11	0	0	0
**day4**	0	0	62	9	0	0
**day5**	0	0	0	2	0	1
**day6**	0	0	0	0	0	1
**day7**	0	0	2	0	0	0
**day8**	0	0	0	2	0	0
**day9**	0	2	1	0	0	0
**day10**	0	0	0	0	0	1
**day11**	0	0	0	0	0	0
**day12**	0	0	1	0	0	0
**day13**	0	1	0	0	0	0
**day14**	0	0	0	0	0	0
**Mean during SS (/day)**	1.4	0.3	5.7	0.9	3.2	0.2
**Relative to pre1d (%)**	0.7	0.0	0.9	0.3	0.6	0.1

Supplementary Table 4. The results of multivariate regression analysis for CORT changes (*top*) and weight changes (*bottom*).

CORT change							
	Estimate	SE	t	*p*	95% Conf. Interval		R^2
**Pitch**	0.0014	0.0014	1.19	0.268	-0.001272	0.0039913	0.151
Intercept	-1.1614	0.7545	-1.54	0.162	-2.901293	0.578408	
**Amplitude**	-0.0003	0.0057	-0.05	0.961	-0.013494	0.0129204	0.000
Intercept	-9.6765	3.7865	-2.56	0.034	-18.40822	-0.9448576	
**Duration**	-0.0015	0.0012	-1.26	0.243	-0.0041021	0.0012021	0.166
Intercept	-1.3574	0.7604	-1.79	0.112	-3.110801	0.395966	

Weight change							
	Estimate	SE	t	*p*	95% Conf. Interval		R^2
**Pitch**	-0.1789	0.3325	-0.54	0.598	-0.88044	0.522657	0.017
Intercept	-1.6994	0.6148	-2.76	0.013	-2.99662	-0.4022379	
**Amplitude**	0.5576	0.4909	1.14	0.272	-0.4781793	1.593443	0.071
Intercept	-2.1954	0.9078	-2.42	0.027	-4.110657	-0.280145	
**Duration**	-0.1112	0.5901	-0.19	0.853	-1.356223	1.133741	0.002
Intercept	-3.6030	1.0911	-3.3	0.004	-5.9051	-1.3010	
