# Supplementary material for: Daily singing of adult songbirds functions to maintain song performance independently of auditory feedback and age
Source: Commun Biol. 2024 May 18;7:598. doi: 10.1038/s42003-024-06311-5 (PMC11102546; doi:10.1038/s42003-024-06311-5)
Supplement: Supplementary file 3 — Description of additional supplementary files [file 42003_2024_6311_MOESM3_ESM.pdf]

Description of Additional Supplementary Files

File name: Supplementary Data 1

Description: The source data behind the major graphs in the paper

File name: Supplementary Audio 1

Description: Pre1d song of a SS-treated bird with relatively large song changes (ID: mgt52mgt41).

File name: Supplementary Audio 2

Description: Post1d song of a SS-treated bird with relatively large song changes (ID: mgt52mgt41).

File name: Supplementary Audio 3

Description: Pre1d song of a SS-treated bird with relatively small song changes (ID: blu80ppl39).

File name: Supplementary Audio 4

Description: Post1d song of a SS-treated bird with relatively small song changes (ID: blu80ppl39)
