# Supplementary material for: Daily singing of adult songbirds functions to maintain song performance independently of auditory feedback and age
Source: Commun Biol. 2024 May 18;7:598. doi: 10.1038/s42003-024-06311-5 (PMC11102546; doi:10.1038/s42003-024-06311-5)
Supplement: Supplementary file 1 — Peer review file [file 42003_2024_6311_MOESM1_ESM.pdf]

nature portfolio

Peer Review File

Daily singing of adult songbirds to maintain song performance independently of auditory feedback and age.

Open Access This file is licensed under a Creative Commons Attribution 4.0 International License, which permits use, sharing, adaptation, distribution and reproduction in any medium or format, as long as you give appropriate credit to the original author(s) and the source, provide a link to the Creative Commons license, and indicate if changes were made. In the cases where the authors are anonymous, such as is the case for the reports of anonymous peer reviewers, author attribution should be to 'Anonymous Referee' followed by a clear attribution to the source work. The images or other third party material in this file are included in the article's Creative Commons license, unless indicated otherwise in a credit line to the material. If material is not included in the article's Creative Commons license and your intended use is not permitted by statutory regulation or exceeds the permitted use, you will need to obtain permission directly from the copyright holder. To view a copy of this license, visit http://creativecommons.org/licenses/by/4.0/.

Reviewers' comments:

Reviewer #1 (Remarks to the Author):

Physical practice of skilled movements is required to maintain high-level performance. In songbirds, singing when no potential mating partner nor other birds against whom the territory has to be defended are around, is thought to serve as practice to maintain a stable song. Adult zebra finches, for instance, sing one highly stereotyped song that remains unchanged over the course of their lives. Here, the authors asked whether this stability depends on song practice. Therefore, zebra finches were prevented from song production by manipulating their posture for two weeks. The song recorded one day before and one day after this manipulation was significantly different with respect to the pitch of harmonic syllables and the duration of the overall song. Specifically, syllables had a lower pitch, and songs were sung faster after the experimental time period. Next, the authors wondered whether the change can be explained by auditory feedback, and the same experiment was conducted with deafened animals. In this group of birds, the authors describe the same changes, which might indicate that the changes are not dependent on auditory feedback. To show that the changes are not an effect of chronic stress induced in the animal by the physical manipulation, cortisol levels were measured, and although a significant increase was detected early on, the effect on song production is not likely to be explained by internal stress.

Although the described effects are reliable, the current conclusion cannot be drawn from this set of experiments alone. One crucial control is missing, which is to restrain the birds with the same methods for a similar amount of time (e.g., during sleep) but allow them to practice during the day. The utilized approach of preventing zebra finches from singing might be too impactful on the acute posture of the animals and might induce temporal muscle pain, soreness, and nerve damage, which might heal over the course of two weeks, and thus song recovers. To exclude this possibility, additional experiments are necessary. Cortisol level measures are not sufficient since they are not a direct readout of the physical stress on muscles and peripheral nerves.

To assess the timeframe of deterioration, additional analysis is necessary. The stability of song in individual birds should be assessed by measuring it over the course of two weeks prior to manipulation. This might answer the question of whether song recovers faster or slower depending on the individual bird's need to practice. Additionally, it is important to determine the progression of recovery, whether it comes back gradually or whether an overnight change can be observed. To achieve this, the song should be analyzed daily after the manipulation has been removed, for the duration of the two weeks. Some of the birds sang despite the perturbation. These data could serve as a good readout of when and how the song deteriorated over the two weeks.

Additionally, it would be best to understand the potential source of change. Do zebra finches need to maintain their muscle tension to preserve their stable singing, or is it the underlying neural circuitry that has to undergo constant activation to maintain its connectivity profile, as suggested by Tschida et al.?

In summary, the manuscript presents some interesting hints towards the necessity of vocal practice to maintain the stable zebra finch song, but a major revision, including new experiments and data analysis, is required to make it suitable for Communications Biology.

Minor comments:

line 7: learn instead of learned
line 10/11 and following: The phrase 'reproductive context' is repeated many times in the abstract and the introduction. To a certain degree, it is misleading since it suggests that the outcome of the following experiment would differ if the birds had been kept with or without a female. It would be interesting to characterize how undirected song, post manipulation, differs from directed singing, and/or whether directed song recovers faster.
line 12: Additionally, the findings should be reported in more detail in the abstract. Specifically, how did the song change, and what do the acoustic and temporal structures imply?
Related to Line 14: An additional experiment would be to prevent intact adult birds from singing and provide them with auditory feedback by playing their own bird song. This would address the question of whether song deterioration is or is not dependent on auditory feedback without impairing the birds.
line 48: In adult zebra finches, undirected singing is not thought to be necessary for refining it but rather a maintenance mechanism to preserve the stereotopy (see also line 80).
line 101: How old were the deafened birds exactly? Degradation of song is correlated with age (Lombardino & Nottebohm). These reversible changes observed in deafened birds speak in favor of an impact of the manipulation itself. (see also line 99). Why did the song of the deafened birds (which were prevented from singing) not deteriorate?
line 128: Is the weight loss correlated with a change in song? A control experiment could be conducted.
line 151: ‘virtually the same’ - by definition, undirected versus directed song is not the same (whereby the first one is more stereotyped and faster).
line 152: How do the authors relate this thought (‘keeping the song in optimal condition’) to the increase in song variability and the idea of vocal exploration? Intrinsic motivation of singing behavior is enhanced after suppression (Kim et al. 2021) – is this in line with what the authors observed? The singing activity after the manipulation should be measured (analogue to Table 2)
line 167: Do zebra finches engage in dawn chorus? The observed deteriorations in song quality in zebra finches cannot solely be related to a ‘warming up the system’ but overnight changes in the neural circuitry have been described (Derégnaucourt et al. 2005).
line 174: What about birds that do sing during the night like mockingbirds, nightingales, certain chats and warblers. Does their song remain stable?
For the broad audience of Communications Biology this study should be put into a broader context: Are there any studies describing vocal output changes in, for instance, subjects that went into silent retreats for some time?
line 198: What is the permit number under which these experiments were authorized? Since the physical manipulation seems to be quite impactful it is concerning whether these experiments would be permitted at a different institute (reproducibility of the data?)
line 207: Animal ethics approval? Songbird cages should be equipped with perches for the welfare of the animals.
line 257 - ‘change’
line 280 – Provide weight measure to assess the severeness of the manipulation.

Reviewer #2 (Remarks to the Author):

This is an interesting study attempting to examine the role of undirected singing as a mechanism to

maintain the structure of song in adult zebra finches. The use of a weight to induce slight postural changes that prevent (or reduce) singing is an attractive approach. There are several issues to be addressed, however.

The results are not well evaluated in relation to existing literature. This goes to the term “passive” song changes, which is used as a descriptive term (song changes without singing) but without reference to any neurobiological model. Instead the paper should consider the potential role of lMAN in driving some of the observations. lMAN, which drives variability during undirected singing in adult birds (the condition studied here), is active during the entire two week period the bird is prevented from singing. Perhaps the bird is trying to sing but fails to phonate, increasing the activity of lMAN during those attempts. In either case, it is not implausible that activity from lMAN may be driving changes in the absence of overt singing. This would be a substantially different interpretation of the results.

There are also two concerns with the data. Supplementary Table 1 shows that six of the 16 birds experienced significant amounts of singing on various days during the two week period of song suppression, and an additional four birds experienced modest amounts of singing, albeit all were below normal levels. Singing with in an abnormal posture may induce abnormal features of song. If singing achieved under conditions designed to prevent it has abnormal features, it is possible that the levels of singing some birds achieved are sufficient to drive changes only observed later given how the data were analyzed. It is essential to analyze these six and perhaps the additional four birds separately, and in a more granual fashion. Were changes relative to baseline observed during the days of singing? That is, are the changes reported here at post 1D release from singing prevention first experienced (in part or in whole) by the bird during the period of singing prevention?

A related point is that the song recordings were optimized to capture song bouts to avoid record calls and cage noises. (Clarify if “bout” refers to a single motif or multiple motifs.) The approach is to use amplitude and time window criteria. Such an approach can fail to record songs that might have been produced at lower amplitude, or songs that were initiated but the motif was truncated earlier than usual, or other adjustments the bird may have made to singing under the challenging experimental conditions. That is, there may have been a great deal more singing the birds experienced than described in Supplementary Table 1. This is troubling as it would undermine the core feature of the study design.

Reviewer #3 (Remarks to the Author):

This interesting study shows that singing restriction over several days causes deterioration of singing performance. Results show that this deterioration reverses quickly with signing and that it does not require auditory feedback – which is very important. It also age independent, which exclude the involvement of age related mechanisms that enable vocal learning in juveniles.

The statistical analysis is mostly okay, but I have one concern: no significant effect of stress was found, but here findings are less convincing because stress hormones level does seem to explain about 25% of the variance, so there might be an issue of power here, which should be discussed. Finally, authors state correctly that the results suggest a peripheral dynamics cause, which is not very

surprising, but perhaps also some neuronal drift – which I think is more interesting. I wonder what strategy can be used in follow-up studies to figure this out?

Finally, I wonder about data availability which is not mentioned: it is now a common practice to deposit the raw data at the time of publication.

Otherwise, the study is well done, and my concerns are fairly minor:

1. The results of testing stress effect seem problematic. Please add a correlation line to all panels in figure 4, and for the ones that are strongly correlated, perform a post hoc power analysis to determine how far are you from showing statistical significance. This is particularly important for panel A where r is 0.52, which is not unlikely to be real.
2. Figure 1: please show an entire song pitch and mean frequency trajectories as in panel D for amplitude. It would also be nice to show if pitch or amplitude changes go together, or alternatively one might be more sensitive than the other.
3. "neural circuitry that generates song structure passively drifts without daily undirected singing" – very interesting: how would you test for that?

Response to reviewers

We thank the reviewers for their reviews. We found their critiques and suggestions very constructive and have addressed them in revision by adding new experimental data and analyses and clarifying the texts including the title and abstract. We find the manuscript greatly improved after incorporating these changes. The following is a point-by-point response to the reviewers' comments.

<u>**Reviewer #1**</u>

Major comments:

Comment 1. Although the described effects are reliable, the current conclusion cannot be drawn from this set of experiments alone. One crucial control is missing, which is to restrain the birds with the same methods for a similar amount of time (e.g., during sleep) but allow them to practice during the day. The utilized approach of preventing zebra finches from singing might be too impactful on the acute posture of the animals and might induce temporal muscle pain, soreness, and nerve damage, which might heal over the course of two weeks, and thus song recovers. To exclude this possibility, additional experiments are necessary. Cortisol level measures are not sufficient since they are not a direct readout of the physical stress on muscles and peripheral nerves.

Response: We have conducted the experiments that the reviewer suggested (restraining the birds with the weights only during the night-time and allowing them to sing during the day). We found that such manipulation does not have significant effects on any of the song features that we examined (syllable pitch, motif amplitude, and motif duration; blue lines and bars in Fig. 1E-J). As the reviewer pointed out, these results exclude the possibility that the SS-induced song changes are mainly caused by posture changes, muscle pain, soreness, or nerve damage, and thus provide evidence supporting the conclusion that the lack of daily singing directly causes the song changes. Please note that the songs of the experimental (singing-suppressed) birds were re-analyzed for the revised manuscript because we found that the previous analyses were not accurate enough, although overall trends of the results are the same as the results presented in the previous manuscript.

Comment 2. To assess the timeframe of deterioration, additional analysis is necessary. The stability of song in individual birds should be assessed by measuring it over the course of two weeks prior to manipulation. This might answer the question of whether song recovers faster or slower depending on the individual bird's need to practice. Additionally, it is important to determine the progression of recovery, whether it comes back gradually or whether an overnight change can be observed. To achieve this, the song should be analyzed daily after the manipulation has been removed, for the duration of the two weeks.

Some of the birds sang despite the perturbation. These data could serve as a good readout of when and how the song deteriorated over the two weeks.

Response: We agree with the reviewer's comment that it is important to determine the progression of song recovery, whether song structure comes back gradually or whether an overnight change can be observed. Accordingly, we have examined the progression of song recovery by analyzing the song features daily after the singing suppression (SS) (Fig. 3; the data from the birds that exhibited relatively large song changes by SS manipulation were analyzed; the songs recorded from post1d to post7d were analyzed because in some birds, songs were not recorded from post8d to post13d). We found that all song features examined (syllable pitch, motif amplitude, and motif duration) gradually recovered toward the pre-SS manipulation levels over multiple days after SS manipulation rather than quickly recovering overnight. Also, all the song features largely returned to the post14d levels at around post7d, although the recovery speeds were quite variable across birds and song features.

As for the song stability prior to SS manipulation, we recorded songs ~1 week before SS manipulation and compared them with the pre1d songs to make sure that overall song structure was stable (i.e. crystallized) in all birds. We did not record songs 2 weeks before SS manipulation as the reviewer suggested because it would raise a significant concern about age-related changes in song structure. Given that the SS manipulation was started at ~100 dph (96 dph at the youngest) for the main experiments (Figs.1-4), it is possible in some birds that songs recorded 2 weeks before SS manipulation (i.e. 82 dph at the youngest) are not fully crystallized and still change over time. Although such age-dependent song changes before SS manipulation could be related to the speeds of song recovery after SS manipulation, given the highly variable degrees of SS-induced song changes themselves across birds (some birds showed almost no changes in song by SS, see Fig. 1E-G), we think that it is difficult to examine the relationship between song stability before SS manipulation and song recovery after SS manipulation (the recovery speed may also depend to some extent on the amount of songs produced each day after the SS period). For this reason, we decided not to conduct a new set of experiments to collect the song data 2 weeks before SS manipulation.

Also, in response to the reviewer's comment on song changes during the SS period, we have analyzed the song features of all the songs produced during the SS period (the data are now presented in Fig. 2A-C of the revised manuscript). Although the number of songs and timing of song production during the SS period were highly variable across birds, we found that both syllable pitch and motif duration decreased gradually over multiple days (on average, over several days for syllable pitch and >1 week for motif duration) to the levels of post1d songs. These gradual changes provide further evidence that SS-induced changes in those features are mainly caused by the reduction of daily singing amounts rather than direct effects of the SS manipulation such as posture changes. In contrast to syllable pitch and motif duration, however, motif amplitude decreased more rapidly, even on the first day of song production during the SS period in all birds, and continued to decrease to the levels even below the post1d song levels in some birds. These rapid and large decreases in motif amplitude are likely to be the direct effects of the SS manipulation (weight attachment) such as posture changes, and therefore raise the possibility that such direct effects lasted even

after the SS period and substantially contributed to the amplitude decreases observed in post1d songs. However, this possibility is clearly ruled out by the results of our new control experiment that the reviewer requested in her/his comment #1: Attaching the weights during night-time but not during daytime for 2 weeks (night-weight control) resulted in no significant changes in song features including motif amplitude (blue lines and bars in Fig. 1E-J). Those birds also showed no significant effects of overnight weight attachment on song performance on the following day (comparison of songs between pre1d and the following day, which were intervened by the first night of weight attachment; Suppl. Fig. 2A). Thus, although the songs that birds managed to produce under the physically restrained condition have abnormally small amplitude, those effects do not last after the SS period nor influence motif amplitude in post1d songs.

Comment 3. Additionally, it would be best to understand the potential source of change. Do zebra finches need to maintain their muscle tension to preserve their stable singing, or is it the underlying neural circuitry that has to undergo constant activation to maintain its connectivity profile, as suggested by Tschida et al.?

Response: We agree with the reviewer that it would be best to understand the potential source of change. As we described in the discussion section (lines 323-333 of the revised manuscript), song changes can be attributable to weakening of the syringeal muscles and/or changes in neural circuits for song production. Indeed, recent findings (Adam et al., Nat Commun 2023) indicate that the absence of vocal practice in zebra finches can result in changes in the physiological parameters of the syringeal muscles. On the other hand, several lines of evidence indirectly support the idea of neural origin of SS-induced song changes: substantial changes in singing-related neural activity in the song motor pathway occur primarily during the nighttime (when birds do not sing), and less frequently during the daytime (Liberti *et al. Nat Neurosci* 2016; Rauske *et al. J Neurosci* 2010); changes in motif duration are observed even in birds that freely sing when the central neural circuits are manipulated (Long & Fee, *Nature* 2008; Kojima *et al. J Neurosci* 2018). To directly examine the contribution of neural circuit changes to SS-induced song changes, it would be necessary to record song premotor activity before and after the SS period from the same neurons and compare the activity patterns. For such experiments, chronic calcium imaging of song premotor activity in a population of neurons would be required. We have added this argument in the Discussion section (lines 338-342).

Minor comments:

Comment 1. line 7: learn instead of learned.

Response: We have changed the text accordingly (line 7).

Comment 2. line 10/11 and following: The phrase 'reproductive context' is repeated many times in the abstract and the introduction. To a certain degree, it is misleading since it

suggests that the outcome of the following experiment would differ if the birds had been kept with or without a female. It would be interesting to characterize how undirected song, post manipulation, differs from directed singing, and/or whether directed song recovers faster.

Response: In response to the reviewer's comment, we have changed the text in the abstract and introduction sections to avoid the repeated use of the phrase 'reproductive context'. As for the experiments of female-directed songs suggested by the reviewer, we agree that it would be interesting to test that idea, but unfortunately, we did not record directed songs from the birds used for collecting the current data. Because investigating the difference in the effects of SS manipulation between undirected song and directed song is not essential to understand the function of daily undirected singing, we have decided not to conduct a new set of experiments for collecting the data of directed song (in addition, our lab lacks the workforce to conduct those experiments because the first author has already left the lab).

Comment 3. line 12: Additionally, the findings should be reported in more detail in the abstract. Specifically, how did the song change, and what do the acoustic and temporal structures imply?

Response: In response to the reviewer's comment, we have added more detailed explanations of song changes in the abstract (lines 11-13). Because the current abstract is already longer than the word limit (150 words), it seems impossible to add more explanations of our findings, unfortunately.

Comment 4. Related to Line 14: An additional experiment would be to prevent intact adult birds from singing and provide them with auditory feedback by playing their own bird song. This would address the question of whether song deterioration is or is not dependent on auditory feedback without impairing the birds.

Response: We appreciate the reviewer's interesting idea of a new experiment. Playback of bird's own song (BOS) during the SS periods would be intriguing and it could prevent song deterioration that is normally caused by SS manipulation. We assume, however, that such a possibility is relatively low because of the following reasons: (1) Multiple lines of evidence suggest that birds evaluate their own song structure only during singing using real-time auditory feedback. For example, both VTA neurons and upstream AIV neurons exhibit fast responses to disruptive auditory feedback presented during singing, but not during non-singing periods (Mandelblat-Cerf et al., eLife 2014; Gadagkar et al., Science 2016; Duffy et al., Cell Rep 2022); (2) Although an off-line process for song maintenance has been proposed (Margoliash & Schmidt, Brain & Language 2009), it does not require playback of BOS to the birds (it is based on spontaneous replays of singing-related activity during sleep); (3) Neurons in multiple nuclei of the song system (HVC, LMAN, Area X etc.) in adult zebra finches exhibit robust auditory responses to BOS playback only during sleep or anesthetized but not during awake (e.g. Dave et al., Science 1998); (4) There is no clear evidence (to our knowledge) showing that playback of BOS to non-singing adult zebra

finches influences the maintenance of their song structure. For these reasons, we expect that BOS playback to singing-suppressed (i.e. non-singing) birds will not significantly influence the SS-induced song changes. Given such a low likelihood of positive results (and the lack of workforce in our lab, as mentioned above), we have decided not to conduct the experiment that the reviewer suggested, although it could be a potential project for future research.

Comment 5. line 48: In adult zebra finches, undirected singing is not thought to be necessary for refining it but rather a maintenance mechanism to preserve the stereotypy (see also line 80).

Response: In response to the reviewer's comment, we have removed the expression "refine song structure" (and related expressions) throughout the manuscript.

Comment 6. line 101: How old were the deafened birds exactly? Degradation of song is correlated with age (Lombardino & Nottebohm). These reversible changes observed in deafened birds speak in favor of an impact of the manipulation itself. (see also line 99). Why did the song of the deafened birds (which were prevented from singing) not deteriorate?

Response: We thank the reviewer for bringing this issue to our attention. The birds were deafened at 98-101 days of age, and most (6/7) birds maintained overall structure of their song during the SS period (we have clarified this information in the revised manuscript [lines 166-168]). As the reviewer pointed out, these results contrast with the substantial song deterioration after deafening in freely-singing birds with similar ages, and indicate that deafening-induced song deterioration requires daily undirected singing. Although these results provide new insight into the mechanisms of deafening-induce song deterioration, we do not highlight this point in our manuscript for the following reasons: (1) The purpose of our deafening experiment is to examine whether the recovery of SS-induced song changes depends on auditory feedback, but not to examine the mechanisms of deafening-induced song deterioration. Because the latter issue is not directly related to the main research question of the current paper, we are afraid that highlighting that issue would disrupt the flow of the paper; (2) To quantify how much deafening-induced song deterioration is blocked by SS manipulation, an additional control experiment in freely-singing deafened birds would be necessary. Given the little relevance of the deafening-induced song deterioration to the main research question of the current paper, we believe that conducting that experiment and quantitative analyses of song deterioration should be done in future research.

Comment 7. line 128: Is the weight loss correlated with a change in song? A control experiment could be conducted.

Response: We have already shown no significant correlations between body weight changes and song changes in the previous version of the manuscript (Fig. 4C, which is now Fig. 6C in the revised manuscript). Although many more control experiments can be

conducted to further strengthen our conclusion, we believe that the new control experiment of nighttime SS manipulation suggested by the reviewer (Comment #1) is sufficient to rule out the possibility that SS manipulation induces song changes mainly by causing physical stress independently of the suppression of undirected singing.

Comment 8. line 151: 'virtually the same' - by definition, undirected versus directed song is not the same (whereby the first one is more stereotyped and faster).

Response: In response to the reviewer's comment, we have modified the text to soften the similarity and clarify the difference between directed and undirected song structure (Line 255-256).

Comment 9. line 152: How do the authors relate this thought ('keeping the song in optimal condition') to the increase in song variability and the idea of vocal exploration? Intrinsic motivation of singing behavior is enhanced after suppression (Kim et al. 2021) – is this in line with what the authors observed? The singing activity after the manipulation should be measured (analogue to Table 2).

Response: We think that this reviewer's comment contains two different (although somehow related) points. The first one is about song variability and the related idea of vocal exploration. As the reviewer pointed out, greater variability of song acoustic structure during undirected singing compared to that during directed singing has been previously reported (e.g., Kao et al. 2005) and it is suggested to serve as motor exploration to optimize song structure (e.g., Tumer & Brainard 2007). This motor exploration-based mechanism of song maintenance requires auditory feedback, by which birds evaluate the quality of ongoing song performance and correct vocal errors. The neural substrates of this song maintenance mechanism have also been intensively studied (e.g., Chen & Goldberg 2020 for review). In sharp contrast, the song maintenance mechanism that we revealed in the current study does not necessarily require auditory feedback, thus appears to be different from the previously well-known song maintenance mechanisms. We have clarified this difference to highlight the novelty of our findings in a new paragraph of the discussion section (lines 259-278).

The second point that reviewer wondered is the relationship of our results with the previous study by Kim et al. 2021, which showed that temporary suppression of undirected singing enhances intrinsic singing motivation (represented by immediate and intense singing). In response to this comment, we have re-analyzed the song data of our SS-treated birds and compared the daily song amounts between pre1d and post1d to examine whether our SS manipulation enhance intrinsic singing motivation. In contrast with the increased singing rate after temporary SS reported by Kim et al. 2021, our results show significant decreases in daily song amounts after the SS period (Suppl. Fig. 4). This discrepancy is likely to be attributable to a large difference in the time course of the experiments between the two studies. In Kim et al. 2021, SS periods were relatively short (up to 10 hr) and ended in daytime hours, and undirected singing behavior was examined immediately after the SS

periods (on the same day). In contrast, we suppressed undirected singing all day (entire daytime period) for 2 weeks (until the evening of the 14th day) and then examined undirected singing behavior on the following day. Because unlike daytime SS, the regular night dark period does not dramatically increase undirected singing motivation (as evidenced by the absence of immediate intense singing in the morning), it is likely that enhanced singing motivation caused by daytime SS is reset by night-time sleep and not maintained overnight into the next day. Additionally, it is possible that our long-term SS manipulation gradually decreases undirected singing motivation over days due to some physiological factors including weakening of the respiratory system responsible for song production. We have described this point in the Discussion section (lines 293-307).

Comment 10. line 167: Do zebra finches engage in dawn chorus? The observed deteriorations in song quality in zebra finches cannot solely be related to a 'warming up the system' but overnight changes in the neural circuitry have been described (Derégnaucourt et al. 2005).

Response: Given the reviewer's comment, we now realized that dawn chorus in zebra finches has not been reported, at least to our knowledge, and that our statement regarding dawn chorus was not appropriate. Therefore, although dawn chorus and the warming up hypothesis in general could be related to the current study to some extent, we have removed the sentences describing these topics from the manuscript.

Comment 11. line 174: What about birds that do sing during the night like mockingbirds, nightingales, certain chats and warblers. Does their song remain stable?
For the broad audience of Communications Biology this study should be put into a broader context: Are there any studies describing vocal output changes in, for instance, subjects that went into silent retreats for some time?

Response: The line 174 in the previous manuscript describes a hypothesized function of the dawn chorus, but in response to the reviewer's comment above (Comment #10), we have decided not to discuss this topic in the revised manuscript. Accordingly, the issue of song maintenance in night-singing birds that the reviewer asked us is not discussed in the revised manuscript either.

As for the second point in the reviewer's comment, we could not find any quantitative studies describing vocal output changes in human subjects that went into silent retreats for some time, but we have instead mentioned a phenomenon in human singers and speakers that their vocal warm-up before performance to optimizes their vocal quality to put our study into a broader context (lines 286-287).

Comment 12. line 198: What is the permit number under which these experiments were authorized? Since the physical manipulation seems to be quite impactful it is concerning

whether these experiments would be permitted at a different institute (reproducibility of the data?)

Response: Our experimental procedure for SS manipulation was reviewed and approved by the Animal Care and Use Committee at our institute, but because we are participating in double-anonymized peer review, we concealed the detailed information including the permit number that may reveal the author's identity, according to the journal's instruction (see https://www.nature.com/documents/nr-double-anonymized-peer-review.pdf). We will add that information in the manuscript after the manuscript is accepted for publication. As for the issue about the reproducibility of our experiments, please note that our procedure for SS manipulation is virtually the same as the one used in a previous study by Hayase *et al*., 2018 (PLOS Biology). Thus, experiments using the same manipulation can be conducted even outside our institute. Those experiments may not be allowed in some countries or institutes because regulations for animal experiments often vary across different countries and institutes.

Comment 13. line 207: Animal ethics approval? Songbird cages should be equipped with perches for the welfare of the animals.

Response: As described above, our experimental procedure for SS manipulation was reviewed and approved by the Animal Care and Use Committee at our institute. Although our standard operating procedures stipulate that the songbird cages for breeding and long-term housing should be equipped with perches, there are no regulations about perches in the cages for short-term experiments. Although detailed regulations may vary across different institutes and countries, we suspect that experiments using cages without perches have been approved at other institutes as well, because there are many studies with chronic recording or manipulation of neural activity using a tether to connect a bird, which can be easily tangled with a perch if it is placed in the cage.

Comment 14. line 257 - 'change'

Response: We have changed the word accordingly (line 417 in the revised manuscript).

Comment 15. line 280 – Provide weight measure to assess the severeness of the manipulation.

Response: The weight changes caused by the SS manipulation were already shown in the manuscript and compared with the song changes (Fig. 4C in the original manuscript and Fig. 6C in the revised manuscript).

Reviewer #2

Comment 1. The results are not well evaluated in relation to existing literature. This goes to the term “passive” song changes, which is used as a descriptive term (song changes without singing) but without reference to any neurobiological model. Instead the paper should consider the potential role of lMAN in driving some of the observations. lMAN, which drives variability during undirected singing in adult birds (the condition studied here), is active during the entire two week period the bird is prevented from singing. Perhaps the bird is trying to sing but fails to phonate, increasing the activity of lMAN during those attempts. In either case, it is not implausible that activity from lMAN may be driving changes in the absence of overt singing. This would be a substantially different interpretation of the results.

Response: We agree with the reviewer that the song changes that we observed following singing suppression (SS) may not be caused by a passive process and could be actively driven by LMAN activity. We have therefore rephrased the term “passive” with different phrases throughout the manuscript (for example, line 15-18). In addition, we have described the possibility of active changes in song structure in the Discussion section (lines 315-316).

Comment 2. There are also two concerns with the data. Supplementary Table 1 shows that six of the 16 birds experienced significant amounts of singing on various days during the two week period of song suppression, and an additional four birds experienced modest amounts of singing, albeit all were below normal levels. Singing with in an abnormal posture may induce abnormal features of song. If singing achieved under conditions designed to prevent it has abnormal features, it is possible that the levels of singing some birds achieved are sufficient to drive changes only observed later given how the data were analyzed. It is essential to analyze these six and perhaps the additional four birds separately, and in a more gradual fashion. Were changes relative to baseline observed during the days of singing? That is, are the changes reported here at post 1D release from singing prevention first experienced (in part or in whole) by the bird during the period of singing prevention?

Response: We agree with the reviewer that it is reasonable to ask whether song changes relative to baseline were observed during the SS period. We have therefore analyzed changes in song features (syllable pitch, motif amplitude and duration) for the songs produced during the SS period (the results are now presented in Fig. 2A-C of the revised manuscript). We found that both syllable pitch and motif duration decreased gradually over multiple days (on average, over several days for syllable pitch and >1 week for motif duration) to the levels of post1d songs (Fig. 2A, C). These gradual changes are consistent with our conclusion that SS-induced changes in those features are mainly caused by the reduction of daily singing rather than direct effects of the SS manipulation (weight attachment) such as posture changes. In contrast to syllable pitch and motif duration, motif amplitude decreased more rapidly, even on the first day of song production during the SS period in all birds, and continued to decrease to the levels even below the post1d song levels in some birds (Fig. 2B). These rapid and large decreases in motif amplitude are likely to be the direct effects of the SS manipulation (weight attachment) such as posture changes,

and therefore raise the possibility that such direct effects lasted even after the SS period and substantially contributed to the amplitude decreases observed in post1d songs. However, this possibility is ruled out by the results of our new control experiment: We attached the weight on bird's neck only during night-time and allowed them to sing freely during daytime for 2 weeks (night-weight control birds), and found no significant effects on song features including motif amplitude (blue lines and bars in Fig. 1E-J). These results suggest that, although the songs that birds managed to produce under the physically restrained condition have abnormally small amplitude, those effects do not last after the weight was removed. This view is also supported by the fact that night-weight control birds showed no significant effects of overnight weight attachment on song performance on the following day (comparison between pre1d and the following day, which were intervened by the first night of weight attachment; Suppl. Fig. 2A). Thus, we conclude that the SS-induced changes in song features observed in post1d songs are mostly attributable to the limited amount of daily singing during the SS period rather than the direct effects of SS manipulation such as posture changes.

We also agree with the reviewer's comment that SS-induced song changes observed on post1d may depend on the amount of singing during the SS period. In response to this comment, we have investigated the relationships between SS-induced song changes and singing amount during the SS period. However, no significant correlations were observed between SS-induced song changes and the number of songs during the SS period for all three song features (Suppl. Fig. 3). These results may reflect the relatively small number of songs produced during the SS period compared to the singing amount before SS (i.e., almost complete suppression of daily singing) in all birds and resulting small variations of song amounts across birds. Although the reviewer suggested to split the data into two sub-groups and analyze them separately, because the singing amount is highly variable across birds and across days during the SS period, we think that it is better to analyze the data of all birds in a gradual fashion as the reviewer also suggested rather than splitting the data into two sub-groups.

Comment 3. A related point is that the song recordings were optimized to capture song bouts to avoid record calls and cage noises. (Clarify if "bout" refers to a single motif or multiple motifs.) The approach is to use amplitude and time window criteria. Such an approach can fail to record songs that might have been produced at lower amplitude, or songs that were initiated but the motif was truncated earlier than usual, or other adjustments the bird may have made to singing under the challenging experimental conditions. That is, there may have been a great deal more singing the birds experienced than described in Supplementary Table 1. This is troubling as it would undermine the core feature of the study design.

Response: Given the decreased amplitude of song motifs produced during the SS period described above (the response to the comment #2), it is possible, as the reviewer pointed out, that the birds produced abnormally quiet songs that our recording system did not detect them during the SS period. We have therefore added a paragraph describing this issue in the Discussion section (lines 308-320). We have argued there that, although our song

recording system was set up to capture sounds that were much quieter than normal zebra finch songs, we cannot exclude the possibility that the birds produced songs with amplitudes that were even below the threshold of our recording system during the SS period. Even if this is the case, however, our results still show that the SS manipulation greatly decreased production of songs that our recording system can detect and resulted in substantial changes in the structure of such detectable songs after the SS period. Because these findings provide evidence that production of such detectable songs is required for maintaining their structure, we believe that our results are still of great significance in understanding the function of daily undirected singing. Although the production of abnormally quiet, whisper-like songs during the SS period is an interesting idea (such songs could be produced, in theory, even by intact, freely singing birds), reliable recordings of such whisper-like songs (even if they really exist) would be technically challenging and required to establish a new experimental setup (for example, attaching a microphone on a bird's head would be necessary to reliably record whisper-like songs). Given such technical difficulty and the uncertainty of the existence of whisper-like songs, we think that testing the whisper-like song idea is beyond the scope of our present study. This argument is supported by a previous study Hayase et al. 2018, in which the authors used basically the same SS manipulation as ours and published the effect of the manipulation in a relatively high-impact journal (PLOS Biology) without testing the possibility of whisper-like songs.

A similar argument applies to the possibility that the birds may have produced abnormally truncated song motifs or made other adjustments in song structure under the SS condition that our recording system could not detect. Although we cannot exclude this possibility, our results still provide evidence that daily production of songs with normal (i.e., untruncated) motif structure is important for maintaining those songs. Additionally, we have examined the effect of SS manipulation on motif truncation by analyzing the last syllable of individual song bouts (we have clarified the definition of "song bout" in response to the reviewer's request, as one or more repetitions of song motifs, separated from other bouts by >0.5 sec silent intervals [lines 122-124 & 366-367]). When the probability of the most typical last syllable was compared between pre1d songs and songs produced during the SS period, we found no significant differences between them (Suppl. Fig. 2B). Because if the SS manipulation induced abnormal truncations of song motifs, probability of the most typical last syllable should decrease from pre1d songs to SS, these results are against the idea of abnormal motif truncations. Moreover, although the reviewer wondered if our song recording system failed to record songs that were initiated but whose motif was truncated earlier than usual, this was unlikely to be the case given the setting of our song recording system. In our experiments, song recording was triggered when the program detected 4 or 5 successive syllable-like sounds (lines 362-363), and most zebra finches' songs start with 3-5 or more introductory notes followed by song motifs that contain more than several syllables. Therefore, the first 4 or 5 sounds of our recordings are mostly introductory notes, and it is likely that most vocalizations that our recording system failed to record are introductory notes (and calls) but not song motifs. For these several reasons above, we believe that our results support the conclusion that the production of undirected songs (with relatively large amplitude and normal structure) is required for maintaining the song performance.

Reviewer #3

Major comments:

Comment 1. The statistical analysis is mostly okay, but I have one concern: no significant effect of stress was found, but here findings are less convincing because stress hormones level does seem to explain about 25% of the variance, so there might be an issue of power here, which should be discussed.

Response: We apologize for a mistake that we have made on Fig. 4B in the original manuscript (Fig. 6B in the revised manuscript). We previously reported an incorrect *r* value. We have now incorporated a new set of statistical analyses to more appropriately address the potential effects of stress on the song parameters that we evaluated. A new table reporting the results of a set of regression analyses can be found in Suppl. Table 4. Additionally, we have included confidence intervals and metrics of effect size, which will allow for a more detailed assessment and interpretation of our results.

Comment 2. Authors state correctly that the results suggest a peripheral dynamics cause, which is not very surprising, but perhaps also some neuronal drift – which I think is more interesting. I wonder what strategy can be used in follow-up studies to figure this out?

Response: To test the idea of neuronal drift, it is necessary to record song premotor activity before and after the SS manipulation from the same neurons. To our knowledge, conventional electrophysiology techniques are not suitable for longitudinal monitoring of neural activity in the same neurons due to frequent drifts of electrodes. One promising approach to overcome this problem is calcium imaging using genetically-encoded calcium indicator and a miniscope implanted on a bird's head (e.g., Liberti et al. 2016; Katlowitz et al. 2018). Calcium recording would allow us to compare song premotor activity between before and after the SS manipulation and examine whether the SS manipulation changes song premotor activity as well as song output. We have added this argument in the Discussion section (lines 338-342).

Comment 3. I wonder about data availability which is not mentioned: it is now a common practice to deposit the raw data at the time of publication.

Response: In response to the reviewer's comment, we have added a statement of data availability after the Materials and Methods section (lines 471-474).

Minor comments:

Comment 1. The results of testing stress effect seem problematic. Please add a correlation line to all panels in figure 4, and for the ones that are strongly correlated, perform a post hoc power analysis to determine how far are you from showing statistical significance. This is particularly important for panel A where r is 0.52, which is not unlikely to be real.

Response: See the answer to the 'Major comment 1' above. Also, as requested, we have now added correlation lines to all panels in Fig. 6B, C.

Comment 2. Figure 1: please show an entire song pitch and mean frequency trajectories as in panel D for amplitude. It would also be nice to show if pitch or amplitude changes go together, or alternatively one might be more sensitive than the other.

Response: Pitch of vocal sounds is generally estimated as the fundamental frequency of a harmonic sound or the mean frequency of a tonal sound, and cannot be accurately defined for other types of sounds such as noisy sounds. Because zebra finch's songs normally include very little tonal sounds, we measured pitch only in harmonic sounds to examine the effect of SS manipulation. Also, even in harmonic sounds, we examined the effect of SS manipulation only in sound segments that had relatively flat pitch trajectories (see the black squares in Fig. 1B), because it is difficult to detect subtle effects on pitch when the pitch trajectories had large frequency modulation (e.g. the first syllable in Fig. 1B). For these reasons, we examined the effects of SS manipulation on pitch only in small portions of individual song motifs (we analyzed 1-3 harmonic segments per motif and averaged the percent changes across the segments). Similar analysis methods have been used in many previous studies that examined subtle changes in syllable pitch of zebra finch songs (e.g., Kao & Brainard 2006, *J Neurophy*; Kojima et al. 2018, *J Neurosci*). Given this pitch analysis on limited segments of a song motif and no analyses of mean frequency, we believe that showing pitch and mean frequency trajectories over the entire song motif is not the best way to represent our results. Although we could show the pitch/mean frequency trajectories for an entire song motif, they will make our results confusing and even invisible, because those trajectories for the unanalyzed segments fluctuate over wide frequency ranges (more than several thousands of Hz; see the figure below), much larger than the pitch changes caused by SS manipulation (only several tens of Hz; see Fig. 1C). We have also clarified our methods of pitch measurements in the Materials and Methods section (lines 394-409).

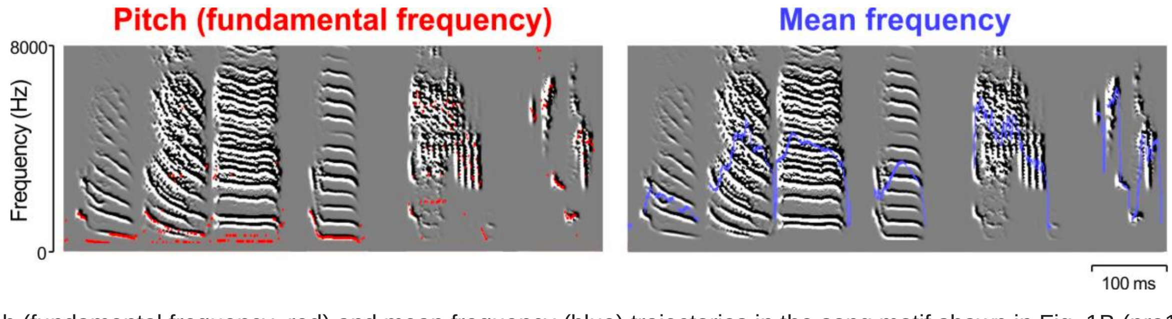

Pitch (fundamental frequency, red) and mean frequency (blue) trajectories in the song motif shown in Fig. 1B (pre1d).

In response to the second point in the reviewer's comment, we examined the relationships between SS-induced changes in the three song features that we analyzed (syllable pitch, motif amplitude, and motif duration). We found significant correlations between motif amplitude and motif duration (Suppl. Fig. 1A), but not between syllable pitch and motif amplitude or between syllable pitch and motif duration (Suppl. Fig. 1B & C,

respectively). These results may reflect the different timescales between syllable pitch and the other song features (motif amplitude and duration).

Comment 3. “neural circuitry that generates song structure passively drifts without daily undirected singing” – very interesting: how would you test for that?

Response: We think that this comment is essentially the same as the same reviewer’s major comment #2 above. Please see our response to that comment.

REVIEWERS' COMMENTS:

Reviewer #1 (Remarks to the Author):

The authors conducted all the requested experiments, demonstrating that song degradation is not caused by the method used to prevent zebra finches from singing, but rather by the lack of song practice. All other concerns were satisfactorily addressed.

Meanwhile, a new paper by Adam et al. has been published in Nature Communication, essentially reporting the same findings while employing a less invasive technique to prevent birds from singing. Given this development, there seems to be a potential diminishment in the novelty of this manuscript. It is currently unclear how the presented work will significantly contribute to advancing our understanding of the essential role of vocal practice in maintaining stable song performance. Consequently, the authors are confronted with the task of refining their study to elucidate the distinct contributions and significance of their new findings, which have not been previously demonstrated.

Reviewer #3 (Remarks to the Author):

All my comments were properly addressed. The MS is now much improved. I have no further comments.
